# The anesthetic sevoflurane induces tau trafficking from neurons to microglia

**DOI:** 10.1038/s42003-021-02047-8

**Published:** 2021-05-12

**Authors:** Yuanlin Dong, Feng Liang, Lining Huang, Fang Fang, Guang Yang, Rudolph E. Tanzi, Yiying Zhang, Qimin Quan, Zhongcong Xie

**Affiliations:** 1 Geriatric Anesthesia Research Unit, Department of Anesthesia, Critical Care and Pain Medicine, Massachusetts General Hospital and Harvard Medical School, Charlestown, MA USA; 2 Department of Anesthesiology, the Second Hospital of Hebei Medical University, Shijiazhuang, P.R. China; 3 Department of Anesthesia, Zhongshan Hospital, Fudan University, Shanghai, P. R. China; 4 Department of Anesthesiology, Columbia University Medical Center, New York, NY USA; 5 Genetics and Aging Research Unit, MassGeneral Institute for Neurodegenerative Disease, Massachusetts General Hospital and Harvard Medical School, Charlestown, MA USA; 6 Rowland Institute at Harvard University, Cambridge, MA USA; 7 Present Address: NanoMosaic, Woburn, MA USA

**Keywords:** Alzheimer's disease, Neuroscience

## Abstract

Accumulation and spread of tau in Alzheimer’s disease and other tauopathies occur in a prion-like manner. However, the mechanisms and downstream consequences of tau trafficking remain largely unknown. We hypothesized that tau traffics from neurons to microglia via extracellular vesicles (EVs), leading to IL-6 generation and cognitive impairment. We assessed mice and neurons treated with anesthetics sevoflurane and desflurane, and applied nanobeam-sensor technology, an ultrasensitive method, to measure tau/p-tau amounts. Sevoflurane, but not desflurane, increased tau or p-tau amounts in blood, neuron culture medium, or EVs. Sevoflurane increased p-tau amounts in brain interstitial fluid. Microglia from tau knockout mice took up tau and p-tau when treated with sevoflurane-conditioned neuron culture medium, leading to IL-6 generation. Tau phosphorylation inhibitor lithium and EVs generation inhibitor GW4869 attenuated tau trafficking. GW4869 mitigated sevoflurane-induced cognitive impairment in mice. Thus, tau trafficking could occur from neurons to microglia to generate IL-6, leading to cognitive impairment.

## Abstract (web summary)

Using their previously developed nanobeam-sensor technology to detect tau protein in the blood, Yuanlin Dong et al. find that the anaesthetic sevoflurane induces trafficking of tau from neurons to microglia in mice. They further see that microglia can take up tau, leading to Il-6 production, overall bringing new insights into the mechanism of sevoflurane-mediated cognitive impairment.

## Introduction

Tau is a microtubule-associated protein that is predominantly found inside neurons and functions in microtubule assembly^1,2^. Tau phosphorylation, aggregation, and spread^3–6^ [reviewed in refs. ^7–9^] all contribute to the neuropathogenesis of age-dependent neurodegeneration in aging brain, including Alzheimer’s disease (AD)^10^ [reviewed in ref. ^11^]. Specifically, tau can spread in brain in a prion-like manner^12–14^ [reviewed in refs. ^15,16^], and tau trafficking can be regulated by 6-O sulfation on heparan sulfate^17^ and low-density lipoprotein receptor-related protein 1^18^. However, key aspects of tau trafficking, including the causes, exit from neurons, transportation, and destination still remain largely to be determined.

Microglia can take up and release tau^19^. Indeed, the microglia obtained from rTg4510 transgenic mice, which overexpress full-length (0N4R) human tau with the P301L frontotemporal dementia mutation in the microglia^20^, contain human tau although they do not express the human tau gene *MAPT*^19^. These data indicate that the tau inside the microglia is from an external source. Additionally, the conditioned culture medium of microglia obtained from rTg4510 mice contains a higher amount of tau and causes more prion-like seeding of tau than the conditioned culture medium of microglia obtained from wild-type littermates^19,20^. These data indicate that microglia can also release tau. However, it remains unknown whether tau can exit from neurons and then enter microglia. Furthermore, the functional consequences of tau trafficking from neurons to microglia are unclear.

Synthetic tau fibrils^21^, mouse brain lysates^22^, and human brain lysates^23,24^, can all induce the spread of tau in mouse brain tissues. Moreover, synthetic tau fibrils can induce the spread of tau in non-neuronal cells^25,26^ and neurons^27,28^.

A novel nanobeam-sensor technology, the label-free assay, was used to overcome the existing technical challenges in tau and phosphorylated tau (p-tau) detection^29,30^. When analytes from the sample bound to the antibodies on the nanobeam-sensor, the spectrum of the nanobeam, as monitored by the laser, shift to the longer wavelength. The nanobeam-sensor is ultrasensitive (up to femtogram/mL), requires a small volume (30 μL)^29,31^, and thus can measure low concentrations of tau and p-tau.

Sevoflurane and desflurane are among the most commonly used inhalation anesthetics in patients. Our previous studies showed that sevoflurane can induce tau phosphorylation and cognitive impairment in mice^32^. Therefore, in the present studies, we used sevoflurane and desflurane as clinically relevant tools and employed nanobeam-sensor as the approach to test a hypothesis that tau and/or p-tau can exit from neurons and enter microglia, which increases the generation of interleukin 6 (IL-6), a pro-inflammatory cytokine, leading to cognitive impairment.

Extracellular vesicles (EVs) are secretory vesicles that are abundant in the body and play important roles in cell-to-cell communication. Elevated EVs concentrations are detectable in cerebrospinal fluid of patients with AD, Parkinson’s disease, prion disease, and amyotrophic lateral sclerosis as compared to healthy control^33,34^. EVs may contribute to the neurotoxicity of β-amyloid (Aβ) and tau^35^. However, it is unknown whether tau and p-tau inside neurons can be transported by EVs to extracellular spaces and microglia. Here, we used tools including nanobeam-sensor technology to measure the amounts of tau and p-tau associated with EVs. We also used GW4869^36–39^, an inhibitor of EVs generation, to assess whether inhibition of EVs generation can attenuate sevoflurane-induced tau trafficking and IL-6 generation in vitro, and cognitive impairment in mice.

## Results

### Elevation of tau in mouse blood following sevoflurane treatment

**Fig. 1:**
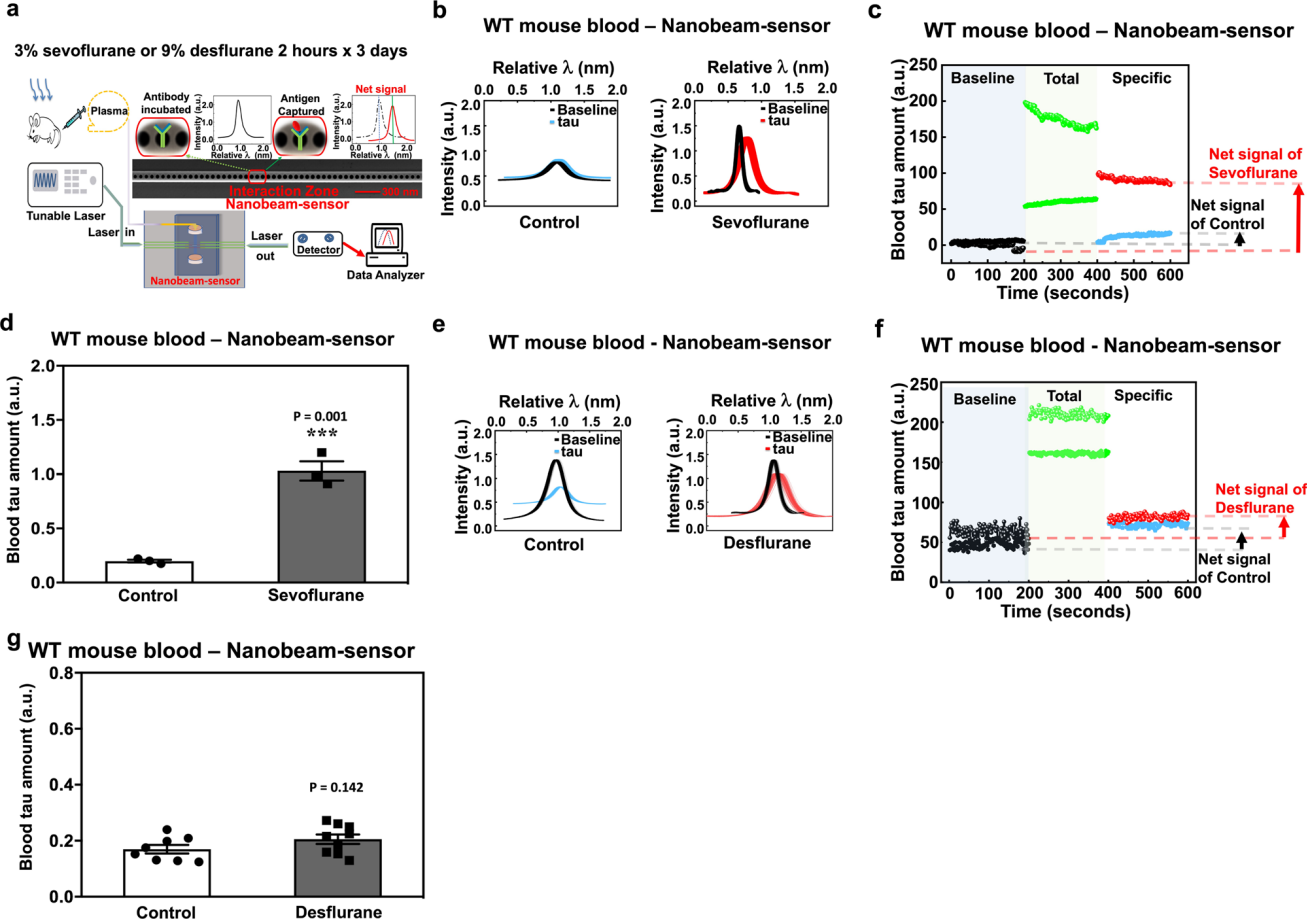
Sevoflurane increased amounts of tau in mouse blood. **a** Diagram of the experimental design. **b** Nanobeam-sensor spectrum data representing tau in the mouse blood following the control condition or sevoflurane. **c** Nanobeam-sensor real-time data representing tau in mouse blood following the control condition or sevoflurane. **d** Quantification of nanobeam-sensor determination of tau in the blood. Nanobeam-sensor spectrum data (**e**), real-time data (**f**), and quantification (**g**) representing tau in the mouse blood following the control condition or desflurane. *N* = 3 or 9 mice (mouse blood study) in each group as demonstrated in each panel of the figure. Student’s *t* test was used to analyze the data presented in Figs. 1d, g. ***P < 0.001. The *P* values refer to the difference of tau amounts in blood between sevoflurane or desflurane and control condition. Error bar indicates standard deviation. WT wild-type.

AD is characterized by the presence of insoluble tau inclusions within neurons^40^. We assessed whether tau or p-tau could be released from neurons following the commonly used anesthetic sevoflurane as part of the in vitro confirmation and further in vivo investigations of the finding from our previous in vivo study^32^. Because it is difficult to measure the amount of tau protein in the blood by conventional methods, we previously developed an innovative ultra-sensitive nanobeam-sensor technology^29,31,41–43^ to accomplish this (Fig. 1a). Our previous study^44^ and present study (Supplemental Fig. 1) demonstrated the specificity and sensitivity of measurement of tau and p-tau by nanobeam-sensor.

The resonance spectra of the nanobeam-sensor were recorded in real-time as the blood sample was applied to the nanobeam-sensor, and after the nanobeam-sensor was washed with PBS to eliminate the non-specific absorptions. The spectrum data (Fig. 1b), the Lorentzian-fit resonance real-time data (Fig. 1c), and the analysis of resonance shifts that quantified tau amounts (Fig. 1d) showed that sevoflurane increased the amount of tau in the blood of the mice as compared to control condition. Desflurane, however, did not increase the amount of tau in the blood (Figs. 1e–g). Sevoflurane treatment (Supplemental Fig. 2a) induced tau phosphorylation, as evidenced by increased amounts of p-tau, but not total tau, in neuron lysates (Supplemental Fig. 2b–d). Desflurane (Supplemental Fig. 2a), however, did not induce tau phosphorylation in neuron lysates (Supplemental Fig. 2e–g).

### Exit of tau and p-tau from neurons following sevoflurane treatment

**Fig. 2:**
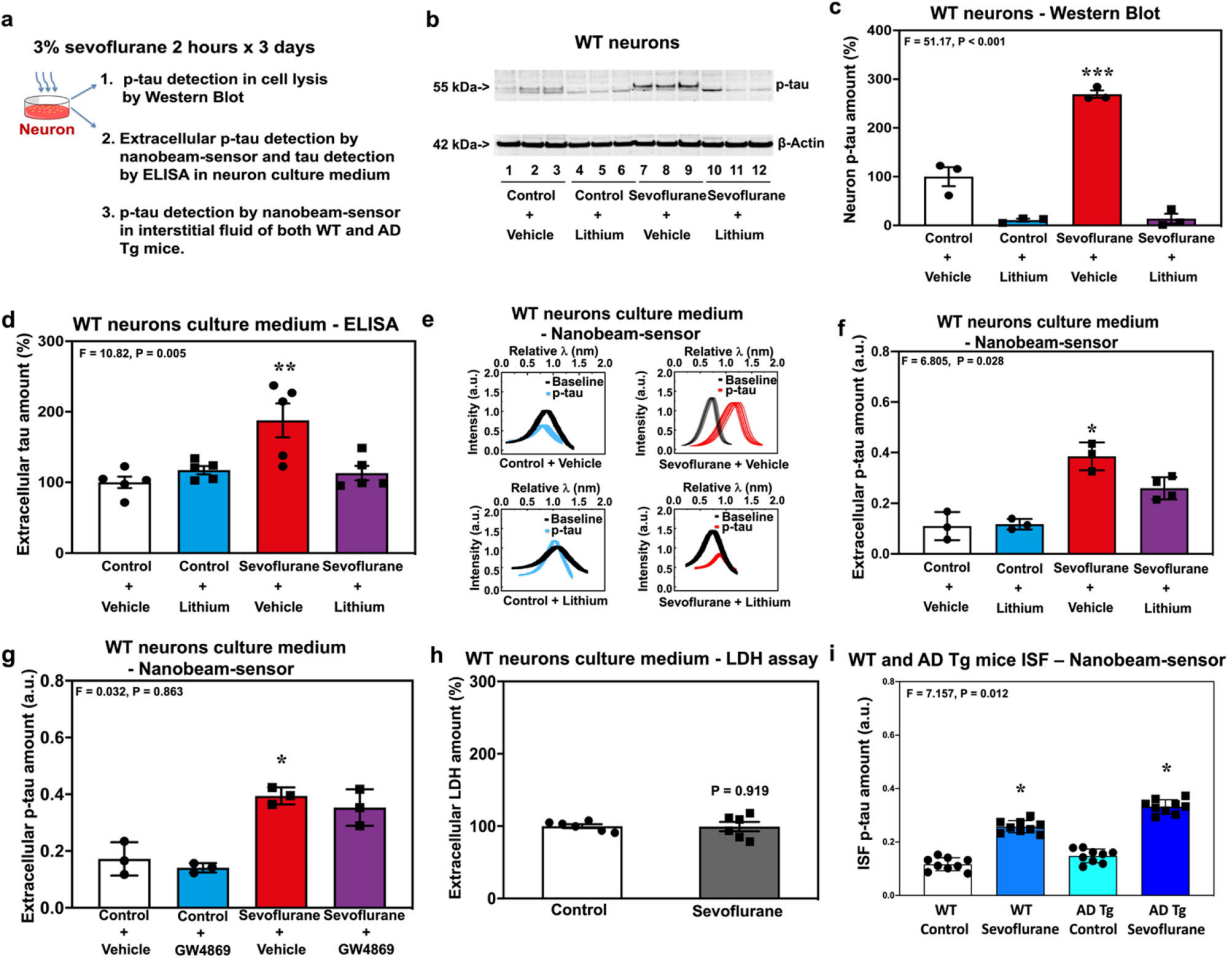
Sevoflurane increased extracellular amounts of tau and p-tau in WT neuron culture medium. **a** Experimental design. **b** Western blot of p-tau in neuron lysates. **c** Quantification of the Western blot in **b**. **d** ELISA of extracellular tau outside of neurons in the neuron culture medium with and without lithium treatment. **e** Nanobeam-sensor spectrum data representing extracellular p-tau outside of neurons in the neuron culture medium. **f** Quantification of the nanobeam-sensor measurement of extracellular p-tau outside of neurons in the neuron culture medium with and without lithium treatment. **g** Quantification of the nanobeam-sensor measurement of extracellular p-tau outside of neurons in the neuron culture medium with and without GW4869 treatment. **h** LDH assay to determine extracellular LDH amounts outside of neurons in the neuron culture medium. **i** Effects of sevoflurane on p-tau amount in brain ISF of both WT and AD Tg mice. *N* = 3 or 5 biologically independent samples (tau and p-tau measurement) as demonstrated in the panel of the figure, 6 biologically independent samples (LDH assay) and 9 mice (ISF p-tau amount) in each group. Two-way ANOVA and post-hoc analysis with Bonferroni were used to analyze the data presented in Fig. 2c, d, f, g, i. The *P* values of the two-way ANOVA refer to the interaction of group (control condition versus sevoflurane), treatment (vehicle versus lithium or GW4869) or mice (WT versus AD Tg mice). The *P* values of post-hoc analysis with Bonferroni refer to the differences in the tau or p-tau amount between control condition and sevoflurane. Student’s *t* test was used to analyze the data presented in Fig. 2h. *P* value refers to the difference in LDH amounts between control condition and sevoflurane. *P < 0.05; **P < 0.01; ***P < 0.001. Error bar indicates standard deviation. P-tau phosphorylated tau, LDH lactate dehydrogenase, WT wild-type, AD Alzheimer’s disease, Tg transgenic, ISF interstitial fluid.

To further determine whether tau can exit from neurons, we set up in vitro studies in which neurons harvested from wild-type (WT) mice were treated with sevoflurane (Fig. 2a). Sevoflurane increased p-tau amounts in neuron lysates and lithium, an inhibitor of tau phosphorylation, attenuated this increase [Fig. 2b (Supplemental Fig. 7) and 2c]. More importantly, sevoflurane treatment also increased the amounts of extracellular tau (Fig. 2d), determined by ELISA; and extracellular p-tau (Figs. 2e, f), determined by nanobeam-sensor, in the neuron culture medium. Similarly, lithium inhibited the sevoflurane-induced increase in extracellular tau or p-tau. Interestingly, GW4869, the inhibitor of EVs generation, did not attenuate the sevoflurane-induced increase in extracellular p-tau (Fig. 2g) in the neuron culture medium. Sevoflurane did not increase extracellular LDH amount, which has a similar molecular weight to tau, in the same neuron culture medium (Fig. 2h). These data suggest that LDH release was not altered with sevoflurane, indicating that neuronal plasma membranes and cell viability were not compromised by the sevoflurane treatment. Finally, we showed that sevoflurane increased p-tau amounts in brain interstitial fluid (ISF) of both WT and AD transgenic (Tg) young mice, with greater increases in the AD Tg mice than the WT mice (Fig. 2i).

We repeated these studies employing desflurane (Supplemental Fig. 3a). Desflurane did not increase the amount of extracellular tau (Supplemental Fig. 3b, ELISA studies) or p-tau (Supplemental Fig. 3c, nanobeam-sensor determination) in the neuron culture medium.

### Presence of tau and p-tau in EVs following sevoflurane treatment

**Fig. 3:**
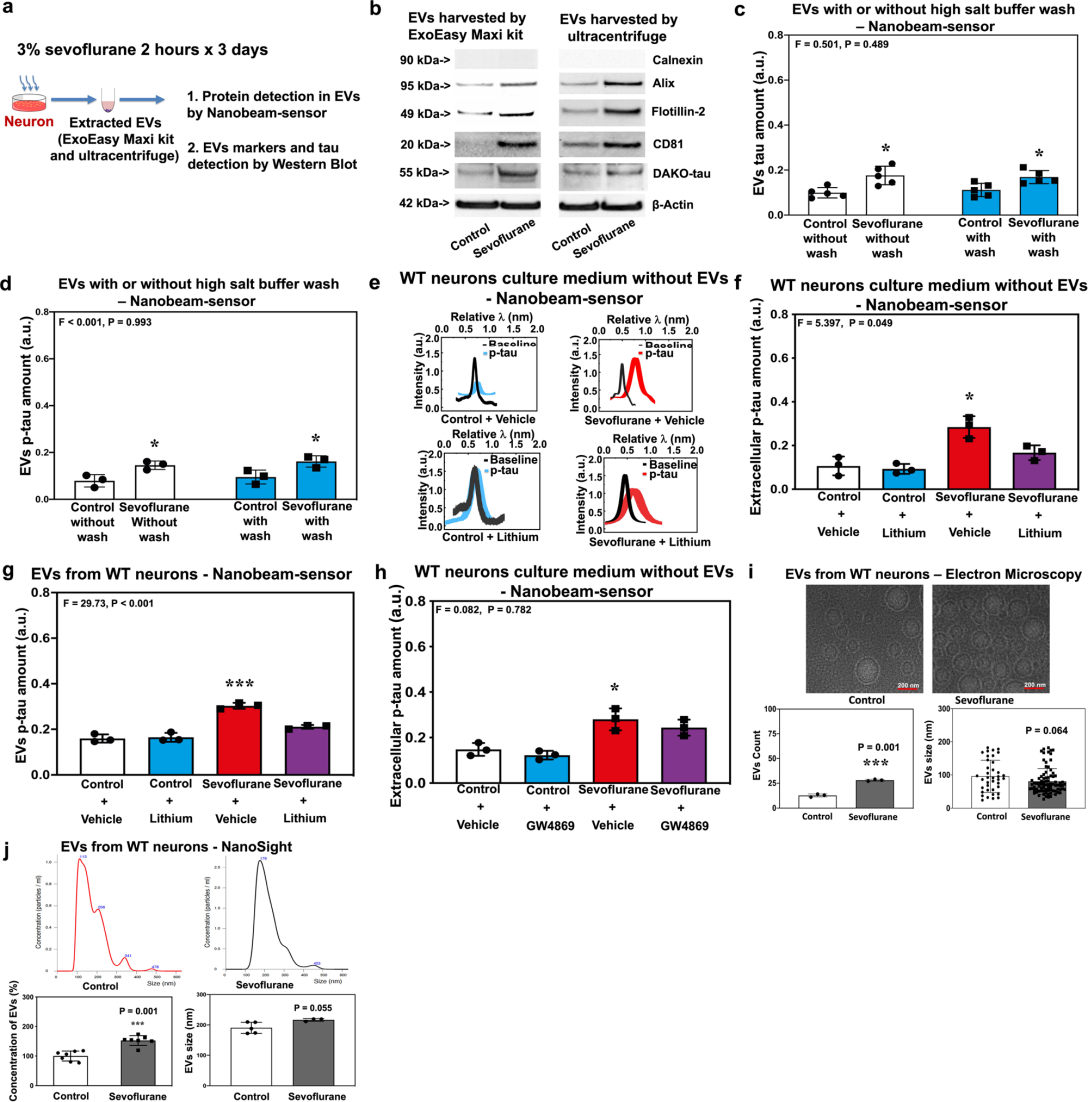
Sevoflurane caused the presence of tau and p-tau in EVs. **a** Experimental design. **b** Effects of sevoflurane on the markers of EVs and tau in EVs. **c** Amounts of tau in EVs with or without treatment of high-salt buffer wash. **d** Amount of p-tau in EVs with or without treatment of high-salt buffer wash. **e** Nanobeam-sensor spectrum data showing extracellular p-tau in WT neuron culture medium without EVs. **f** Quantification of nanobeam-sensor measurement of p-tau in **e**. **g** Nanobeam-sensor measurement of p-tau in the EVs extracted from neurons with and without lithium treatment. **h** Nanobeam-sensor measurement of extracellular p-tau amounts in neuron culture medium after extraction of EVs with and without GW4869 treatment. **i** Electron microscope measurement of the count and size of EVs. **j** NanoSight analysis of the concentration and size of EVs. Two-way ANOVA and post-hoc analysis with Bonferroni were used to analyze the data presented in Figs. 3c, d, f, g, h. Student’s *t* test was used to analyze the data presented in Fig. 3i, j. The *P* values of the two-way ANOVA refer to the interaction of group (control condition versus sevoflurane), method (with versus without high-salt buffer wash), or treatment (vehicle versus lithium or GW4869). The *P* values of post-hoc analysis with Bonferroni refer to the differences in the tau or p-tau amounts between control condition and sevoflurane. *P*-values of the Student’s *t* test (Fig. 3i, j) refer to the difference of count, concentration, and size between control condition and sevoflurane. *P < 0.05, and ***P < 0.001. *N* = 3 biologically independent samples in each group in Fig. 3d, f, g, h, i (left panel) and Fig. 3j (right panel); *N* = 5 biologically independent samples in each group in Fig. 3c, j (right panel); *N* = 7 biologically independent samples in each group of Fig. 3j (left panel); *N* = 38 EVs in the control group and *N* = 84 EVs in the sevoflurane group of Fig. 3i (right panel). Error bar indicates standard deviation. P-tau phosphorylated tau, EVs extracellular vesicles, WT wild-type, EM electron microscopy.

Next, we determined the effects of sevoflurane or desflurane on the amount of p-tau in EVs (Fig. 3a and Supplemental Fig. 4a). We first showed that sevoflurane increased amounts of Alix, Flotillin-2, and CD81, the positive markers of EVs, but not calnexin, the negative marker of EVs^45^ [Fig. 3b (Supplemental Fig. 8)]. Sevoflurane also increased tau amounts in EVs, detected by Western blot using antibody detecting DAKO-tau (Fig. 3b)^46^.

Next, nanobeam-sensor analysis showed that sevoflurane increased amounts of tau (Fig. 3c) and p-tau (Fig. 3d) in EVs, which were harvested with or without high-salt buffer wash. Nanobeam-sensor analysis showed that sevoflurane increased the amount of extracellular p-tau in neuron culture medium without EVs (after extraction of EVs) and lithium inhibited this increase (Fig. 3e, f). Nanobeam-sensor analysis demonstrated that sevoflurane increased the amount of p-tau in the lysates of EVs harvested from WT neurons, and lithium attenuated this increase (Fig. 3g). By contrast, nanobeam-sensor analysis showed that desflurane did not increase the amount of p-tau in lysates of EVs harvested from WT neurons (Supplemental Fig. 4a, b). Consistently, in neuron culture medium without EVs, GW4869 did not attenuate the sevoflurane-induced increase in the amount of p-tau (Fig. 3h). Electron microscopy (Fig. 3i) and NanoSight (Fig. 3j) showed that sevoflurane increased the count, but not size, of EVs. Western blot showed that sevoflurane increased the amount of Alix and flotillin-2, the markers of EVs, in WT mouse brain tissues (Supplemental Fig. 4c–e).

**Fig. 4:**
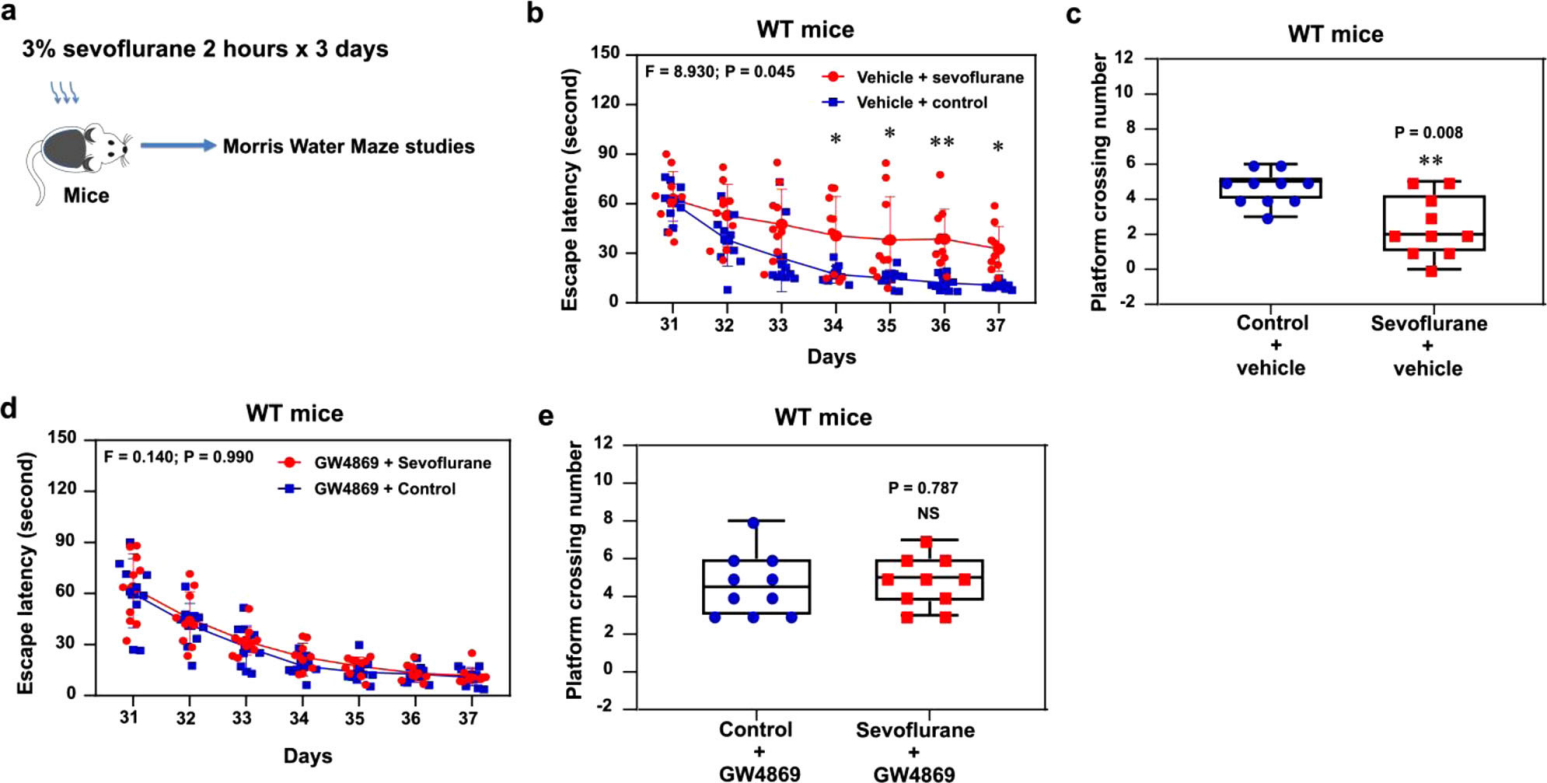
GW4869 mitigated the sevoflurane-induced cognitive impairment in mice. **a** Experimental design. Effects of sevoflurane on cognitive function in mice pre-treated with vehicle (**b**, **c**) in MWM. Effects of sevoflurane on cognitive function in the mice pre-treated with GW4869 (**d**, **e**) in MWM. *N* = 10 mice in each group. Two-way ANOVA with repeated measurement and post-hoc analysis with Bonferroni were used to analyze the data presented in Fig. 4b, d. The *P* values of the two-way ANOVA refer to the interaction of group (control condition versus sevoflurane) and days (day 31 to 37). The *P* values of post-hoc analysis with Bonferroni refer to the differences in escape latency between sevoflurane and control condition on each day. The Mann–Whitney U test was used to analyze the data presented in Fig. 4c, e, the *P* values refer to the difference of the number of platform crossing between control condition and sevoflurane. *P < 0.05 and **P < 0.01. The box indicates median (50^th^ percentile), the first quartile (25^th^ percentile), and the third quartile (75^th^ percentile) of the number of platform crossing; the upper and lower bar indicates the minimum and maximum of platform crossing number. Error bar indicates standard deviation. MWM Morris water maze.

Finally, we found that sevoflurane treatment induced cognitive impairment (Fig. 4a) in the mice pre-treated with vehicle (Fig. 4b, c), but not in the mice pre-treated with GW4869 (Fig. 4d, e).

### Tau and p-tau were taken up by microglia following treatment with sevoflurane-conditioned neuron culture medium

**Fig. 5:**
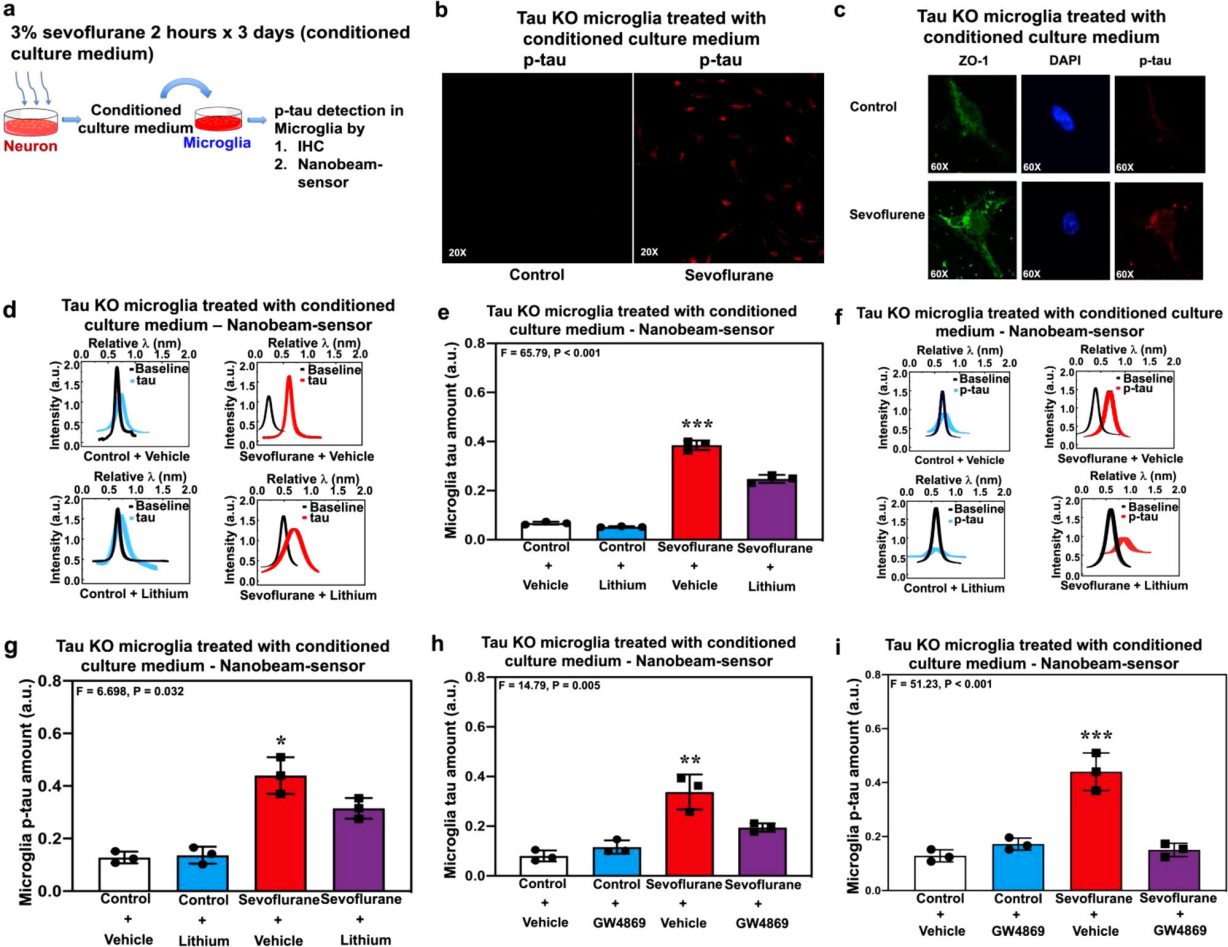
Sevoflurane-conditioned neuron culture medium caused the appearance of tau and p-tau in tau KO microglia. **a** Experimental design. **b** Immunohistochemistry showing the appearance of p-tau in a group of tau KO microglia following the treatment of sevoflurane-conditioned neuron culture medium. **c** Immunohistochemistry showing the appearance of p-tau in a single tau KO microglia following the treatment of sevoflurane-conditioned neuron culture medium. **d** Nanobeam-sensor spectrum data representing tau in the lysates of tau KO microglia following the treatment of sevoflurane-conditioned neuron culture medium with and without lithium. **e** Quantification of nanobeam-sensor measurement of tau. **f** Nanobeam-sensor spectrum data representing p-tau in the lysates of tau KO microglia following the treatment of sevoflurane-conditioned neuron culture medium with and without lithium. **g** Quantification of nanobeam-sensor measurement of p-tau. **h** Nanobeam-sensor measurement of tau in the lysates of tau KO microglia following the treatment of sevoflurane-conditioned neuron culture medium with and without GW4869. **i** Nanobeam-sensor measurement of p-tau in the lysates of tau KO microglia following the treatment of sevoflurane-conditioned neuron culture medium with and without GW4869. *N* = 3 biologically independent samples in each group. Two-way ANOVA and post-hoc analysis with Bonferroni were used to analyze the data presented in Fig. 5e, g, h, i. The *P* values of two-way ANOVA refer to the interaction of group (control condition versus sevoflurane) and treatment (vehicle versus lithium or GW4869) on the amounts of tau or p-tau. The *P* values of the post-hoc analysis with Bonferroni refer to the difference on the amounts of tau or p-tau between the control condition and sevoflurane. *P < 0.05, **P < 0.01, ***P < 0.001. Error bar indicates standard deviation. P-tau phosphorylated tau, KO knockout.

We next asked whether the tau or p-tau exiting from neurons could be taken up by microglia (Fig. 5a and Supplemental Fig. 5a). We treated tau KO microglia, which do not contain any tau or p-tau, with the sevoflurane-conditioned neuron culture medium, which contained tau and p-tau. We first confirmed that the cells used in the study were indeed microglia using flow cytometry (Supplemental Fig. 5b) and immunohistochemistry (Supplemental Fig. 5c). Immunohistochemistry showed the presence of p-tau in tau KO microglia (Fig. 5b, c) following treatment with the sevoflurane-conditioned neuron culture medium. Next, nanobeam-sensor technology demonstrated that the culture medium from neurons treated with sevoflurane plus vehicle increased the amounts of tau (Fig. 5d, e) and p-tau (Fig. 5f, g) in the lysates of tau KO microglia. However, the culture medium from neurons treated with sevoflurane plus lithium caused less increase in the amount of tau (Fig. 5d, e) and p-tau (Fig. 5f, g) in the lysates of tau KO microglia. These data demonstrated that the tau or p-tau exiting from neurons could be taken up by microglia. Finally, we found that the sevoflurane-conditioned neuron culture medium increased the amounts of tau (Fig. 5h) and p-tau (Fig. 5i) in the tau KO microglia, but the conditioned culture medium of neurons treated with sevoflurane plus GW4869 (inhibitor of EVs generation) caused less increases in the amounts of tau (Fig. 5h) and p-tau (Fig. 5i) in the tau KO microglia. Immunohistochemistry also showed that conditioned culture medium of neurons treated with sevoflurane, but not desflurane, might increase the amount of p-tau inside tau KO microglia (Supplemental Fig. 5d). Finally, nanobeam-sensor analysis demonstrated that lysates from tau KO microglia did not show increases of p-tau amounts when treated with culture medium from desflurane-treated neurons (Supplemental Fig. 5e).

### Enhanced generation of IL-6 in neurons plus microglia following sevoflurane treatment

**Fig. 6:**
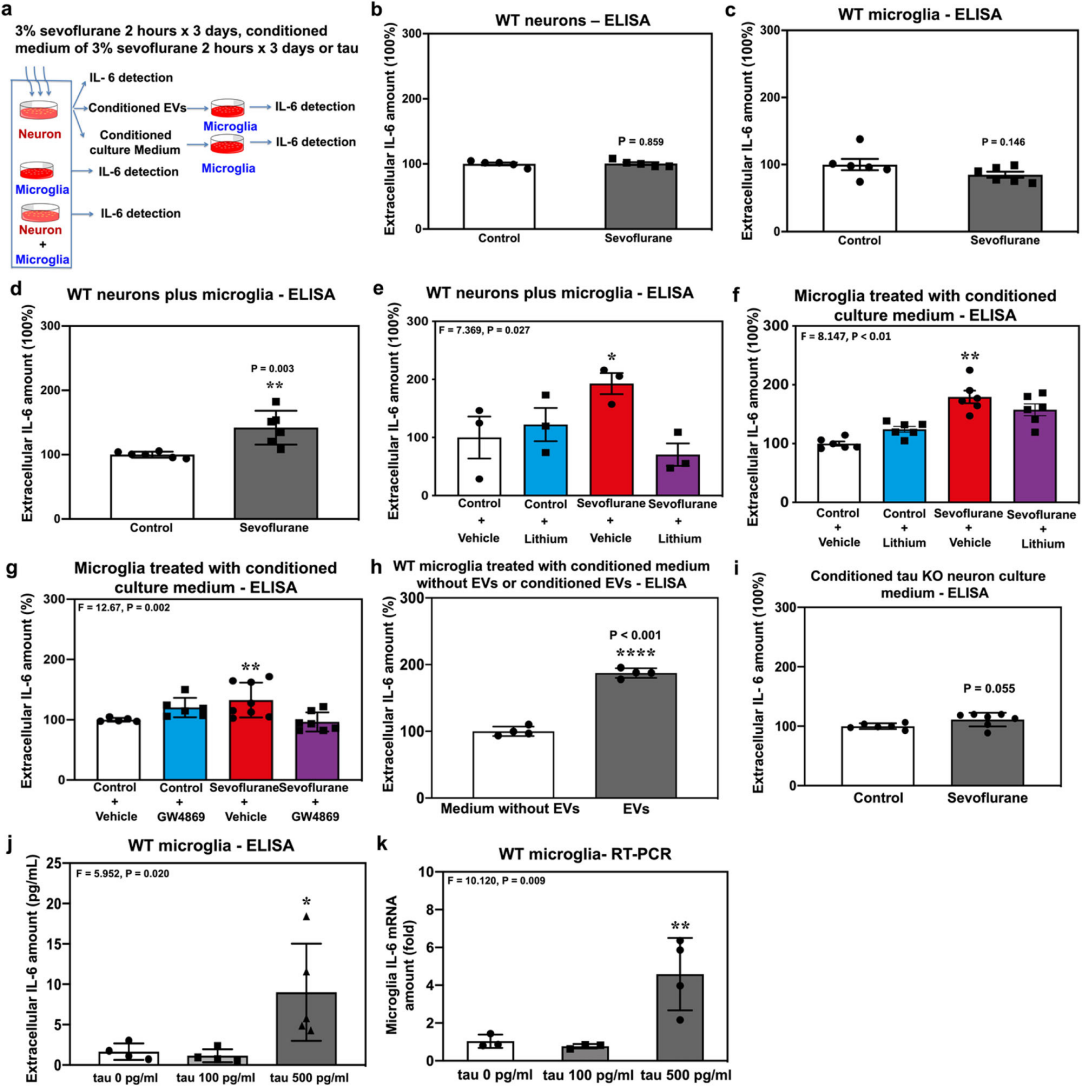
Sevoflurane increased extracellular IL-6 amounts in the culture medium of neurons plus microglia. **a** Experimental design. **b** Extracellular IL-6 amounts in the WT neuron culture medium. **c** Extracellular IL-6 amounts in the WT microglia culture medium. **d** Extracellular IL-6 amounts in the culture medium of WT neurons plus WT microglia. **e** Extracellular IL-6 amounts in the culture medium of WT neurons plus WT microglia with and without lithium treatment. **f** Extracellular IL-6 amounts in the culture medium of WT microglia treated with sevoflurane-conditioned neuron culture medium with and without lithium. **g** Extracellular IL-6 amounts in the culture medium of WT microglia treated with sevoflurane-conditioned neuron culture medium with and without GW4869. **h** Comparison of extracellular IL-6 amounts in the culture medium of WT microglia treated with the conditioned EVs specifically extracted from the conditioned medium versus the same conditioned medium without those extracted EVs. **i** Effects of sevoflurane conditioned tau KO neuron culture medium on extracellular IL-6 amount of microglia. **j** Extracellular IL-6 amounts in WT microglia treated with tau protein. **k** IL-6 mRNA expression in WT microglia treated with tau protein. *N* = 3 to 8 biologically independent samples in each group as demonstrated in each panel of the figure. Two-way ANOVA and post-hoc analysis with Bonferroni were used to analyze the data in Fig. 6e–g, the *P* values refer to the interaction of group (control condition and sevoflurane) and treatment (vehicle versus lithium or GW4869) on IL-6 amounts. One-way ANOVA was used to analyze the data in Fig. 6j, k, the *P* values of one-way ANOVA and the post-hoc analysis with Bonferroni refer to the difference in IL-6 amounts from 0, 100, and 500 pg/ml of tau. Student’s *t* test was used to analyze the data in Fig. 6b, c, d, h, i, the *P* values refer to the difference in IL-6 amounts between the control condition versus sevoflurane. *P < 0.05, **P < 0.01, ****P < 0.001. Error bar indicates standard deviation. P-tau phosphorylated tau, KO knockout, EVs extracellular vesicles, WT wild-type.

We assessed the functional consequences of tau trafficking from neurons to microglia by treating neurons, microglia, or neurons plus microglia with sevoflurane (Fig. 6a). ELISA showed that sevoflurane did not increase the amounts of extracellular IL-6 in neurons alone (Fig. 6b) or microglia alone (Fig. 6c). However, sevoflurane significantly increased the amounts of extracellular IL-6 in the culture medium of neurons plus microglia (Fig. 6d).

Inhibition of tau phosphorylation with lithium attenuated the sevoflurane-induced increase in extracellular IL-6 amounts in the culture medium of neurons plus microglia (Fig. 6e). Moreover, the culture medium of WT microglia alone showed the increased extracellular IL-6 amounts when the microglia were treated with the sevoflurane-conditioned neuronal culture medium, and such increase in IL-6 was attenuated by lithium (Fig. 6f) or GW4869 (Fig. 6g). The conditioned EVs specifically extracted from the conditioned medium caused greater increases of extracellular IL-6 amount as compared to the conditioned medium without those extracted EVs (Fig. 6h). Notably, the conditioned culture medium from the tau KO neurons treated with sevoflurane did not increase extracellular IL-6 amounts of microglia as compared to control condition (Fig. 6i). Moreover, treatment of WT microglia with exogenous tau increased extracellular IL-6 amounts (Fig. 6j) and the expression of IL-6 mRNA (Fig. 6k).

Activated NF-kB includes the activation of p65, which translocates to the nucleus and binds to the promoter region of multiple genes including cytokine genes to generate IL-6^47^. Thus, we determined the effects of sevoflurane on p65 expression in the nuclei of WT microglia (Supplemental Fig. 6a). Immunohistochemistry showed that treatment with sevoflurane-conditioned neuron culture medium for 30 or 120 min increased the expression of p65 in the nuclei of microglia (Supplemental Fig. 6b–d). Western blot demonstrated that sevoflurane increased the ratio of phosphorylated p65 to p65 (Supplemental Fig. 6e, f) and the ratio of phosphorylated STAT3 and STAT3 (Supplemental Fig. 6g, h) as compared to control condition. These data suggest that sevoflurane could increase IL-6 via, at least partially, activation of NF-kB or STAT3 pathways.

The source data regarding the quantifications of Figs. 1 to 6 and Supplemental Figs. 1 to 6 can be found in the Supplemental data 1 within the Supplemental information of this manuscript.

## Discussion

Anesthetics can affect tau pathology and cognitive function^32^. Thus, we used anesthetic sevoflurane and desflurane as clinically relevant tools to study tau trafficking in the present study. The data obtained from in vitro and in vivo experiments suggest that tau can exit from neurons upon phosphorylation, travel through both EVs and non-EVs routes, and then enter microglia, leading to generation of IL-6.

Sevoflurane-induced increase of blood tau amounts demonstrated the potential for tau to exit from neurons (Fig. 1). However, tau can also be released from other organs, including the lungs or other non-neuron cells^48^. Our in vitro studies revealed that sevoflurane, but not desflurane, induced tau phosphorylation at serine 202 and threonine 205 residues in neurons (Supplemental Fig. 2), and simultaneously increased extracellular tau and p-tau amounts in the neuron culture medium (Fig. 2). Sevoflurane also enhanced p-tau amounts in the brain ISF of young mice with greater increase in the AD Tg mice than the WT mice (Fig. 2). These results demonstrated the AD-relevant in vivo findings of tau trafficking following sevoflurane treatment. The tau and p-tau could appear in EVs from WT neurons (Fig. 3) and microglia from tau KO mice (Fig. 5). Importantly, lithium, an inhibitor of tau phosphorylation^49^, and GW4869, an inhibitor of EVs generation^50^, attenuated the sevoflurane-induced increases in p-tau amounts in microglia. GW4869 also attenuated the sevoflurane-induced cognitive impairment (Fig. 4). Taken together, these data suggest tau trafficking occurs through a neuron-EVs-microglia route or neuron-non-EVs-microglia route, which are associated with tau phosphorylation, following certain condition (sevoflurane) since such tau trafficking did not occur upon treatment with desflurane, another commonly used inhalation anesthetic. Importantly, sevoflurane treatment did not cause the exit of LDH, which has a molecular weight similar to that of tau (Fig. 2). These data indicate that the tau exited from neurons was not due to passive transport of cellular contents owing to neuron breakage.

Notably, both tau and p-tau appeared in the neuron culture medium, EVs and microglia (Figs. 2, 3 and 5). These data suggest that both tau and p-tau can exit from neurons. However, the findings that lithium, the inhibitor of tau phosphorylation, attenuated the sevoflurane-induced increases in both tau and p-tau in neuron culture medium, EVs and microglia demonstrated that such increases were dependent, at least partially, on tau phosphorylation.

Interestingly, anesthetic sevoflurane, but not desflurane, induced tau trafficking in the present study. The exact reason of such difference is not clear at the present. Compounds with lower chemical bond-dissociation energy levels are more unstable and thus more easily interact with proteins^51,52^, generating free radicals. Sevoflurane and desflurane may have different chemical bond-dissociation energy owing to their different molecular structures. Thus, we hypothesized that desflurane could be less toxic (e.g., promoting less or no tau trafficking) due to less generation of free radicals compared with sevoflurane.

EVs, a heterogeneous group of vesicles released by cells under physiological and pathological conditions^53–55^, have been classified as ectosomes, microvesicles, microparticles, exosomes, oncosomes, apoptotic bodies, and more based on their origin^50^. Exosomes are released from intracellular compartments known as multi-vesicular bodies, and their biogenesis occurs through endosomal sorting complexes required for transport machinery (ESCRT)-dependent and ESCRT-independent pathways. The ESCRT-independent pathway is regulated by neutral sphingomyelinases. GW4869 is a potent neutral sphingomyelinase inhibitor that can prevent exosome generation and release^36,50,56,57^. Thus, the finding that GW4869 mitigated the sevoflurane-induced changes in tau trafficking and cognitive impairment in mice suggests a role of EVs-associated tau trafficking in AD neuropathogenesis and cognitive function. However, GW4869 attenuated the sevoflurane-induced increase in p-tau only in microglia, but not in conditioned medium nor medium without EVs. This was because the p-tau (and/or tau) could exist in different locations: outside and inside of EVs. Thus, the amounts of the p-tau in the conditioned medium were not affected by GW4869, the inhibitor of EVs generation. These data suggest that tau or p-tau associated with EVs may not appear in the neuron culture medium but may appear in the microglia. The conditioned EVs specifically extracted from the conditioned medium were able to increase more extracellular IL-6 amounts as compared to the same conditioned medium but without those extracted EVs (Fig. 6h). These results suggest that the tau and p-tau associated with EVs may have dominant roles on the sevoflurane-induced IL-6 increase in microglia than the tau or p-tau not associated with EVs. Therefore, we hypothesized that the tau or p-tau associated with EVs were most readily internalized by microglia. The future study will use the established system to test this hypothesis, including the identification of the type of tau or p-tau (e.g., free tau or p-tau versus tau or p-tau inside vesicle versus tau or p-tau fragment) that is most readily internalized by microglia. Notably, GW4869 and other compounds reported to prevent EVs release including calpeptin, manumycin A, Y27632, D-pantethine, and imiparamine, have other effects in addition to their inhibitory effects on the generation and release of EVs^50^.

Sevoflurane increased the amounts of positive markers, but not negative marker, of EVs, and the amounts of tau (detected by DAKO-tau antibody) in EVs harvested by either ExoEasy Maxi kit or ultracentrifuge method (Fig. 3). These data suggest that both methods can effectively harvest EVs in the present study. Moreover, high-salt buffer (0.8 M) did not significantly affect the amounts of tau and p-tau associated with EVs (Fig. 3).

We did not lyse blood, brain ISF, and culture medium prior to the measurement of tau or p-tau by the nanobeam technology. Therefore, the tau or p-tau measured in the blood, brain ISF, or culture medium was likely the tau or p-tau that was not in vesicles. Nevertheless, we will use the established system to systemically identify the specific type of tau and p-tau that can be released by anesthetic sevoflurane in the future.

Lithium, the inhibitor of tau phosphorylation, attenuated the sevoflurane-induced increases in extracellular p-tau amount in neuron culture medium, but GW4869 did not attenuate the sevoflurane-induced increases in extracellular p-tau amount in the WT neuron culture medium with (Fig. 2g) or without EVs (Fig. 3h). These data demonstrated that the exit of tau from neurons was dependent, at least partially, on tau phosphorylation, but not totally on EVs generation. There could be other mechanisms of tau release from neurons in addition to EVs.

We used tau KO microglia in the study because the presence of tau or p-tau inside tau KO microglia confirms the uptake of tau or p-tau. The findings that sevoflurane-conditioned (containing increased amounts of tau and p-tau), but not desflurane-conditioned neuron culture medium (not containing increased amounts of tau and p-tau), rendered the presence of tau or p-tau inside the tau KO microglia (Fig. 5 and Supplemental Fig. 5) suggest that the tau or p-tau exited from neurons can enter to microglia and imply that spreading of tau from neurons to microglia is exacerbated by sevoflurane, but not desflurane, treatment.

Sevoflurane only increased the amounts of extracellular IL-6 in the culture medium of neurons plus microglia but not in neurons or microglia alone (Fig. 6). Thus, the combination of neurons and microglia was necessary to generate IL-6, which further supported the hypothesis that tau trafficking occurred from neurons to microglia, leading to IL-6 generation. Moreover, the sevoflurane conditioned culture medium of tau KO neurons did not increase IL-6 amount, further suggesting that the increase in IL-6 amounts was dependent on tau, consistent with the findings in previous studies^32,58^. Finally, sevoflurane-conditioned neuron culture medium induced activation of NF-kB signaling, which was attenuated by lithium (Supplemental Fig. 6). These data suggest that NF-kB signaling could be one of the underlying mechanisms by which sevoflurane increased IL-6 amounts.

Microglia, the resident immune cells of the brain, play an important role in neuroinflammation and AD neuropathogenesis^59–61^. Specifically, microglial activation has been shown to promote tauopathy, including tau phosphorylation and aggregation in vitro and in mice^62–67^. On the other hand, tauopathy also activates microglia^60,68^. These findings strongly suggest that the interaction of tau and microglia plays a critical role in the propagation of tauopathy throughout the central nervous system^35,67^. Specifically, a study by Hopp et al. demonstrated that microglia can uptake, process, and release tau in mice and in vitro^19^. Consistently, the current study indicated that tau or p-tau could move into microglia (Fig. 5 and Supplemental Fig. 5). Moreover, our findings suggest that tau can exit from neurons into the extracellular space via a mechanism associated with tau phosphorylation. The exited tau could “swim” and travel into microglia with the assistance of EVs or non-EVs pathway, leading to generation of IL-6 and cognitive impairment.

Our previous studies^44^ and the present study (Supplemental Fig. 1) have shown the specificity and sensitivity of detection of tau and p-tau by using nanobeam technology. However, it is still important to compare our newly established nanobeam technology with other technology in detecting tau and p-tau, including Simoa^69^ and mass spectrometry^70^ in the future.

The study has several limitations. First, other proteins, including β-amyloid protein, may also enter the microglia to increase IL-6 generation. The current data cannot exclude this possibility. However, the findings that lithium attenuated the exit of tau from neurons, the transportation of tau via EVs, the appearance of tau in tau KO microglia, and the generation of IL-6 suggest that tau phosphorylation was involved in the tau trafficking from neurons to microglia, leading to the generation of IL-6. Second, the data obtained from the current study could not distinguish whether the tau or p-tau inside the microglia was passively transported by EVs or was actively taken up by microglia. Future studies to reveal the underlying mechanisms are warranted.

In conclusion, using inhalation anesthetics sevoflurane and desflurane as clinically relevant tools and nanobeam-sensor technology as the detection method, we demonstrated that tau or p-tau could exit from neurons to the extracellular space through mechanisms associated with tau phosphorylation. The exited tau or p-tau could travel with the assistance of EVs or non-EVs pathway, and enter microglia, activating the NF-kB signaling pathway and leading to IL-6 generation and cognitive impairment. These data demonstrated a neuron-EVs-microglia or neuron-non-EVs-microglia pathway of tau trafficking, which has functional consequences on IL-6 generation and cognitive impairment. This work will likely lead to more research on anesthesia, tau trafficking, and AD neuropathogenesis.

## Methods

### Mice, anesthesia treatment, and brain tissue harvest

The animal protocol was approved by the Standing Committee on Animals at Massachusetts General Hospital, Boston, MA (protocol 2006N000219). Efforts were made to minimize the number of animals used. The manuscript was written according to ARRIVE guidelines. Adult wild-type (WT) mice (C57BL/6 J, Strain#: 000664), tau knockout (KO) mice (Strain#: 007251), and AD transgenic (Tg) mice (5XFAD, Strain#: 034848) were purchased from The Jackson Laboratory (Bar Harbor, ME). Female and male WT or AD Tg young mice at postnatal day 6 were obtained by breeding in our lab. Mice were randomly assigned to the following groups: (1) control; (2) control plus DMSO or saline (vehicle for GW4869 or lithium); (3) control plus GW4869 or lithium; (4) sevoflurane or desflurane; (5) sevoflurane plus DMSO or saline; (6) sevoflurane plus GW4869 or lithium. Mice were anesthetized with 3% sevoflurane or 9% desflurane plus 60% oxygen for 2 h daily on postnatal days 6, 7, and 8^32,71^, or 6, 8, and 10^72^ (only for mouse brain ISF harvest experiments). Control mice received 60% oxygen at an identical flow rate in identical chambers. We continuously monitored sevoflurane, desflurane, and oxygen concentrations using a gas analyzer (Dash 4000; GE Healthcare, Milwaukee, WI). Anesthesia chamber temperature was monitored and controlled by a feedback-based system with a DC temperature control system (World Precision Instruments Inc, Sarasota, FL), which automatically adjusted to keep the mouse body temperature at 37 °C ( ± 0.5 °C) via a warming pad placed under the chamber. For intervention studies, we treated mice with GW4869 (10 mg/kg, dissolved in 10% DMSO and corn oil at 0.45 µg/µL; Cat#: D1692, Sigma, St. Louis, MO)^50^ through intraperitoneal administration 30 min before each sevoflurane treatment on postnatal days 6, 7, and 8. Mice were decapitated at the end of the sevoflurane or desflurane treatment session on postnatal day 8, and cortex tissues were harvested.

### Microdialysis of mouse brain interstitial fluid (ISF)

Postnatal day 10 female and male WT or AD Tg mice were used in the studies after the anesthesia with 3% sevoflurane on postnatal day 6, 8, and 10. The ISF was collected by microdialysis^50^. Specifically, microdialysis probe (Harvard Apparatus, Cambridge, MA) was cannulated into brain (cortex area) of the mice under 3% sevoflurane anesthesia for 10 min on postnatal day 10. On the day of microdialysis, the microdialysis probe was checked for no leakage and the membrane of the probe was submerged in 70% ethanol for two seconds. Artificial cerebrospinal fluid (1.3 mM CaCl_2_, 1.2 mM MgSO_4_, 3 mM KCl, 0.4 mM KH_2_PO_2_, 25 mM NaHCO_3_, 122 mM NaCl, pH = 7.35) was used for the exchange with ISF. We performed the microdialysis and collected ISF at 1.5 ul per minute for 60 min by using the pump (Harvard Apparatus). The collected ISF was analyzed by nanobeam-sensor for amounts of p-tau (tau-PS202/PT205).

### Nanobeam-sensor technology

We established a label-free nanobeam technology^29,30^ to detect tau and p-tau in blood, ISF, neuron culture medium, EVs, and microglia. Note that the blood, ISF, and neuron culture medium was not lysed before the measurement of tau or p-tau by the nanobeam technology. The establishment and mechanism of the nanobeam-sensor were described in detail in previous publications^29,30^. We used an anti-tau 5 antibody (Cat# ab80579, Abcam, Cambridge, MA) and a phospho-tau (Ser202, Thr205) monoclonal antibody (AT8) (MN1020, ThermoFisher Scientific, Waltham, MA) to capture tau and p-tau, respectively. We used a home-made single channel microfluidic device (100 µm by 50 µm) for sample delivery. The peak of each spectrum was obtained using a Lorentzian fitting algorithm^29^.

### Morris water maze (MWM)

Morris water maze tests were performed on postnatal day 31 to 37, with four trials per day for seven days^32,71^. Each group had 10 mice. Specifically, we tested the mice in MWM four trials per day from postnatal day 31 to 37 as the reference training. The escape latency was recorded. At the end of the reference training (postnatal day 37), the platform was removed from the pool and the mouse was placed in the opposite quadrant. Each mouse was allowed to swim for 90 s and the numbers the mouse swam to cross the platform area were recorded as platform crossing number.

### Mouse blood harvest

After the last sevoflurane or desflurane anesthesia on postnatal day 8, each mouse was removed from the chamber. Anesthesia was maintained via a cone device. We exposed the heart and collected blood with a 1 microliter syringe.

### Neuron harvest

Primary neurons were harvested^73,74^. We euthanized WT mice with the gestation stage of day 15 by using carbon dioxide. We then pull out the embryos and decapitate them in a 100-mm dish of phosphate-buffered saline. We put the head on the top of a 100-mm dish and dissected out the cortices, removed meninges. Finally we placed the neurons into another 100-mm dish of phosphate-buffered saline for culture.

### Microglia harvest

Primary microglia were prepared from cerebral cortices of 2-day-old neonatal tau KO mice or C57Bl/6 J WT mice^75,76^. After removing the meninges, cortical tissues were digested with 0.25% trypsin-EDTA for 30 min at 37 °C, then gently mechanically triturated in DMEM/F12 (Cat#: 11320-033, ThermoFisher) with 10% fetal bovine serum (FBS) (Cat#: 26140-079, ThermoFisher). Mixed cortical cells were filtered through a nylon membrane with 100 µm pore size and plated in 6-well plates in the same complete culture medium, which was replaced every 3–4 days until confluence was achieved after about 14 days. Microglia were isolated from mixed glial cultures via mild trypsinization (0.25% trypsin-EDTA diluted 1:4 in DMEM/F12). Incubation of the mixed glial culture with trypsin for 15–25 min resulted in detachment of an intact layer of cells, while microglia remained attached to the bottoms of the wells. The microglia were then detached from the culture plate with 0.25% trypsin-EDTA and re-seeded with an equal number of cells per plate, and allowed to rest overnight before treatment with sevoflurane, sevoflurane-conditioned culture medium, or sevoflurane-conditioned neuron EVs. Purity of the isolated microglia was determined using flow cytometry and immunohistochemistry.

### Co-culture of neurons plus microglia

On day 5 after seeding primary cortical neurons, the harvested microglia were isolated, detached, and seeded in each neuron plate at a 10:1 ratio of neurons to microglia. We co-cultured neurons and microglia overnight before sevoflurane or desflurane treatment on days 6, 7, and 8.

### Treatment of cultured cells with sevoflurane or desflurane

Equal numbers of neurons, microglia, or neurons plus microglia were seeded in six-well plates that were placed in a sealed plastic box and kept in a 37 °C incubator. On days 6, 7, and 8 after seeding of the neurons, we used an anesthesia machine to deliver 21% O_2_, 5% CO_2_, and 3% sevoflurane or 9% desflurane to the neurons for two hours daily for three days. We changed the culture medium right before the treatments. A gas analyzer was used to continuously monitor the concentrations of delivered carbon dioxide, oxygen, and sevoflurane or desflurane. In the interaction studies, 0.5 mM glycogen synthase kinase 3β (GSK3β) inhibitor lithium (Cat#: 499811, Sigma) diluted in saline and 10 μM GW4869 diluted in DMSO were administrated to the neurons one hour before the sevoflurane treatment.

### EVs isolation

Same amount of neuron (e.g., 30 millions/sample for Western Blot analysis, 10 millions/sample for nanobeam study, and 4 million/sample for the microglia treatment) was used in various experimental groups of EVs studies. Neuron culture medium supernatants were carefully collected after sevoflurane or desflurane treatment with or without lithium (0.5 mM) or GW4869 (10 μM) treatment. *Ultracentrifugation:* EVs were directly isolated from the supernatants through differential centrifugation. The neuron culture medium was first centrifuged at 300 × *g* for 10 min at 4 °C to remove free cells, then at 3000 × *g* for 20 min at 4 °C to remove cellular debris, and finally at 10,000 × *g* for 30 min at 4 °C to remove free organelles. Supernatant was transferred to a clean quick-seal ultracentrifuge tube and ultracentrifuged at 100,000 × *g* for 2 h at 4 °C (70.Ti Beckman rotor, Beckman Coulter, Jersey City, NJ) to attain an EVs-enriched pellet. We measured EVs positive markers Alix, Flotillin-2 and CD81, and EVs negative marker Calnexin in the harvested EVs obtained from 30 millions/sample neurons. We also collected the supernatants from culture medium without EVs to measure the amounts of tau and p-tau. *ExoEasy Maxi Kit method:* EVs were also isolated using an exoEasy Maxi Kit (Cat#: 76064, Qiagen, Germantown, MD) according to the accompanying protocol. This method can specifically and completely remove the conditioned neuron culture medium (containing tau and p-tau) from EVs. EVs quantity and size were determined by an electron microscope (EM) and Nanosight. The ultracentrifugation method was used for the measurement of the amounts of EVs negative marker Calnexin, and EVs positive markers Alex, Flotillin 2 and CD81, and tau; for the collection of the conditioned culture medium without EVs for the determination of the amounts of tau and p-tau, and for the isolation of the conditioned EVs. The ExoEasy Maxi Kit method was used in the measurement of the amounts of EVs negative marker Calnexin, and EVs positive markers Alex, Flotillin 2 and CD81, and tau; for the isolation of conditioned EVs for the determination of tau and p-tau amounts in EVs, electron microscopy; and for the NanoSight studies determining EVs number and size. EVs were also treated with high-salt buffer (0.8 M), made from 10 X PBS (including 1.37 M NaCl, pH 7.4), for 30 min at 4 degree^77^, which determined whether tau or p-tau detected in EVs was adhered to the outside of EVs.

### Treatment of microglia with sevoflurane- or desflurane-conditioned culture

We used sevoflurane- or desflurane-conditioned neuron culture medium to determine whether extracellular tau or p-tau could enter microglia. Following treatment with sevoflurane, desflurane, or control conditions, neuron culture medium was collected and used to treat tau KO microglia for six hours to determine whether tau or p-tau could enter tau KO microglia.

### Treatment of microglia with the conditioned medium, the conditioned EVs, and the conditioned medium without EVs for the measurement of extracellular IL-6

We treated microglia with the conditioned medium for 6 h. We also split full conditioned medium into the part of conditioned EVs and the part of conditioned medium without the EVs. Specifically, the medium in two cell culture dishes with 20 million neurons in 6 ml conditioned medium per dish was centrifuged as described in the section of EVs isolation. The supernatant (12 ml) was harvested as the conditioned medium without EVs and the pellets (the isolated EVs) were suspended into 12 ml fresh medium as the conditioned EVs. Finally, we treated same number of microglia with the conditioned EVs or the conditioned medium without EVs in same volume of medium for 6 h.

### Treatment of microglia with tau

We treated microglia with tau protein at 100 pg/mL and 500 pg/mL (Cat#: ab151872, Abcam) to determine the effect of tau on the amount of released extracellular IL-6 and IL-6 mRNA expression.

### Brain tissue, neuron, microglia, and EVs lysis, and protein quantification

Harvested brain tissues were homogenized on ice using a lysis buffer including immunoprecipitation buffer (Mammalian Protein Extraction Reagent, Cat# 78501, Thermo Scientific) plus protease inhibitor cocktail (Cat# 11836170001, Sigma). The neurons treated with anesthetics were detergent-extracted on ice using the same lysis buffer and lysates were collected with a cell scraper. Microglia were treated with sevoflurane-, desflurane-, or control-conditioned neuron culture medium. After removing the conditioned culture medium and washing the samples twice with 1 x PBS, the microglia were incubated in 0.05% trypsin-EDTA (Cat#: 25300-054, ThermoFisher) for one minute to remove the possible binding of tau to the cell surface. The trypsin was quickly removed, and the microglia were washed two more times with 1 x PBS, then detergent-extracted on ice in the same volumes of the lysis buffer. Specifically, we re-suspended the isolated EVs (obtained from the same amounts of neurons) with equal volumes of lysis buffer, and waited 10 min to allow complete lysis for the detection of Alix, Flotillin-2, CD81, Calnexin and tau by Western blot, and tau and p-tau by nanobeam-sensor. Lysates were collected and centrifuged for 15 min at about 19,000 × g. The total amount of protein was quantified using the Pierce protein assay kit (Cat#: 23225, ThermoFisher).

### Reverse transcriptase-polymerase chain reaction (RT-PCR)

RNA was harvested and isolated from microglia after tau protein treatment. Real-time RT-PCR was performed using the QuantiTect SYBR green real-time PCR kit (Cat#: 204243, Qiagen) to detect the amounts of IL-6 mRNA. The catalog number of the IL-6 primers was QT00098875 (Qiagen).

### Western blot

Total tau was detected using the anti-tau 5 antibody (Cat# ab80579, 55 kDa, 1:1000, Abcam). Tau-PS202/PT205 antibody (AT8) (Cat# MN1020, 55 kDa, 1:200, ThermoFisher) was used to detect tau phosphorylated at serine 202 and threonine 205 residues. Alix antibody (Cat#: sc53538, 95 kDa, 1:200, Santa Cruz, Dallas, TX), anti-Flotillin-2 antibody (Cat#: 3436 S, 49 kDa, 1:1000, Cell Signaling Technology, Danvers, MA), anti-CD81 antibody (20 kDa, Cat#: ab109201, 1:1,000, Abcam), anti-Calnexin antibody (90 kDa, Cat#: 2433 s, 1:1000, Cell Signaling Technology), and anti-DAKO-tau antibody (55 kDa, Cat#: A002401-2, 1:200, Agilent technologies, Santa Clara, CA) were used to detect Alix, Flotillin-2, CD81, Calnexin and DAKO-tau, respectively, in EVs. NF-kB p65 antibody (6H7) (65 kDa, Cat#: MA515563, Thermo Fisher Scientific, WB: 1:1000), Phospho-NF-kB P65 (SER536) antibody (65 kDa, Cat#: MA515181, Thermo Fisher Scientific, WB: 1:1000), Phospho-Stat3 (Tyr705) antibody (86 kDa, Cat#: 9131 S, Cell Signaling Technology, WB: 1:1000), and Stat3 antibody (86 kDa, Cat#: 9139 S, Cell Signaling Technology, WB: 1:1000) were used to detect p65, p-p65, p-STAT3, and STAT3 in Western blot analysis, respectively. β-actin antibody (42 kDa, Cat#: A5441, 1:5000, Sigma), detecting non-targeted protein β-actin, served as a loading control. Western blot quantification was performed as described by Xie et al.^78^. Signal intensity was analyzed using ChemiDoc XRS + with Image Lab 5.0 software (Bio-Rad, Hercules, CA).

### Lactate dehydrogenase (LDH) assay

Amounts of LDH released into the cell culture medium were used to detect cell membrane integrity or cell viability. Extracellular LDH amounts were assessed using a cytotoxicity detection kit (Cat#: 11644793001, Sigma) according to the manufacturer’s instructions.

### Enzyme-linked immunosorbent assay (ELISA)

*Tau ELISA*. We used the mouse tau immunoassay ELISA kit (Cat# KMB7011, ThermoFisher) to determine extracellular tau amounts in the neuron culture medium after sevoflurane or desflurane treatment with or without lithium. Briefly, we added 50 μL of standard diluent buffer and 50 μL of standard or sample to each well. Plates were mixed and incubated for 2 h at room temperature and washed four times with 1 X wash buffer. We added 100 μL of 1 X tau biotin conjugate solution to each well and incubated for one hour, then washed four times with 1 X wash buffer. After incubation of the substrate solution for 30 min, the reaction was stopped with 100 μL stopping buffer per well. We determined the optical density of each well using a SpectraMax i3x Multi-Mode microplate reader (Medical Device, San Jose, CA) at 450 nm. *IL-6 ELISA*. The mouse IL-6 immunoassay kit (Cat #: M6000B, R&D Systems, Rochester, MN) was used to determine the amount of IL-6 in the culture medium of neurons, microglia, neurons plus microglia, and the medium of microglia treated with sevoflurane- or desflurane-conditioned culture medium or neuron EVs. Briefly, a monoclonal antibody specific for mouse IL-6 was coated onto microplates. We added 50 μL of assay diluent RD1-14, and then added 50 μL of standard or samples to the center of each well. Wells were mixed and incubated for 2 h at room temperature and washed five times with wash buffer. Then, 100 μL of mouse IL-6 conjugate was added to each well and incubated for another 2 h and the mixtures were washed five times. After the wells were incubated in 100 μL of substrate solution in a dark room for 30 min, the reaction was stopped with the stop solution. Determination of the optical density of each well was set at 450 nm and corrected at 570 nm.

### Flow cytometry

Isolated microglia were treated with 0.25% trypsin-EDTA and collected for flow cytometry. Cells were counted and 10^6^ cells in 45 μL PBS were treated with 5 μl mouse Fc block (anti-CD16/CD32 antibody in the form of 2.4G2, Cat#: 553142, BD Biosciences, San Jose, CA) for 10 min at room temperature to inhibit nonspecific binding. Cells were stained with fluorochrome-conjugated anti-CD11b-PE antibody (Cat#: 101208, BioLegend, Dedham, MA) and Toll-like receptor 4 (TLR4)-FITC antibody (Cat#: ab45126, Abcam) for 30 min in the dark at room temperature. We washed cells twice with 4 ml PBS and re-suspended them in 0.5 mL PBS. A total of 10^5^ cells were acquired per sample using a LSRFortessa X-20 cell analyzer (BD Biosciences), and data were analyzed using FlowJo software (BD Biosciences).

### Immunohistochemistry

Tau KO microglia were treated with sevoflurane- or desflurane-conditioned neuron culture medium. At the end of treatment, microglia seeded onto FluoroDishes were washed with 0.05% trypsin followed by PBS. We fixed the microglia with 4% paraformaldehyde in 0.1 M PBS at pH 7.4 for 15 min on ice. For staining, microglia were incubated with biotin-conjugated AT8 (Cat# MN1020B, 1:50, ThermoFisher), ZO-1 (Cat#: 402200, 1:500, ThermoFisher), or IBA1 (Cat#: 019-19741, Fujifilm Wako, Richmond, VA) antibodies overnight at 4 °C, followed by streptavidin-conjugated Alexa Fluor 594 (Cat#: S32356, 1:500, ThermoFisher), Alexa Fluor 488 donkey anti-rabbit IgG (Cat#: A21206, 1:500, ThermoFisher), and Alexa Fluor 546 goat anti-rabbit IgG (Cat#: A11010, 1:500, ThermoFisher) antibodies for 1 h at room temperature in the dark. Microglia were analyzed in Fluoroshield mounting medium with DAPI (Cat#: ab104139, Abcam) under a 10 X objective of a KEYENCE BZ-9000E all-in-one fluorescence microscope (Keyence Corporation of America, Itasca, IL) for IBA1 study. To detect p-tau inside tau KO microglia, we employed a Nikon Eclipse Ti confocal microscope (Nikon, Melville, NY) with 20 X and 60 X objective lenses and photographs were taken.

### NF-kB activation

WT microglia seeded in FluoroDishes were treated with sevoflurane-conditioned neuron culture medium with or without lithium for 30 min or 120 min. Microglia were fixed with 4% paraformaldehyde and stained with NF-kB p65 antibody (Cat#: sc-109, 1:200, Santa Cruz), followed by Alexa Fluor 546 goat anti-rabbit IgG (1:500, ThermoFisher). Quantification of immunohistochemical dishes (9–14 fields in each treatment group) was performed by unbiased, double-blinded stereological counts. The only inclusion criterion was clear microscopic observation of the cell body; exclusion criteria included a dirty background and broken cell bodies. Immunohistochemical photomicrographs were captured at 200 X magnification with a confocal microscope. Percentage of NF-kB p65 positive cells was determined per random field. Moreover, the ratios of phosphorylated p65 to p65 and phosphorylated STAT3 to STAT3 were also assessed to detect potential NF-kB activation^79^.

### Nanoparticle tracking analysis (NTA)

Nanoparticle tracking can characterize the size and number of EVs. Purified EVs from control and sevoflurane conditions were resuspended in 500 μl of sterile double-filtered PBS with a 0.22-um filter. EVs solution was measured for nanoparticle content using a NanoSight LM10 system (NanoSight Ltd, Amesbury, UK) configured with a 405-nm laser and a high-sensitivity CMOS camera. The detection threshold was 7, blur and minimum expected particle size was set to “auto”. Temperature was recorded manually and did not exceed 25 °C. Three videos of 60 s duration were recorded per sample. Data were analyzed using NanoSight NTA 3.2 Software.

### Transmission Electron Microscope (EM) studies of EVs

EVs samples were fixed 1:1 with 2% glutaraldehyde (v/v; Sigma Aldrich) for 30 min. A fixed sample of 5 µL was pipetted onto a 400-mesh copper grid and incubated for 10 min. Images were acquired through the Tecnai F20 (FEI) microscope system (ThermoFisher Scientific). Images were obtained from the average of two 700 mini-second acquisitions with 2048 × 2048 resolution and Imaging Solutions software (Olympus, Tokyo, Japan). To find a magnification showing as many EVs as possible on a single image with sufficient detail to distinguish EVs morphological features, we evaluated images at 100,000×, 58,000×, and 32,000× magnifications. Higher magnification images appeared to have better background quality. We defined EVs diameters of ≥20 nm with sufficient contrast for picture selection.

### Statistics and reproducibility

Based on our previous studies, we determined that 10 mice per group for behavioral studies, and 3-9 samples per group for biochemistry studies including nanobeam-sensor, Western blot, ELISA, LDH assay, NanoSight, RT-PCR, EM, and immunohistochemistry would provide sufficient statistical power. We present data from biochemistry studies and Morris water maze escape latency as mean ± SD; and platform crossing numbers from the Morris water maze are presented as median and interquartile range. Interaction between time and group factors was determined using two-way ANOVA with repeated measurements to analyze the difference in learning curves (based on escape latency) between mice in the control and sevoflurane treatment groups in the Morris water maze test. Post-hoc analysis with Bonferroni was used to compare the escape latency between mice in the control and anesthesia treatment groups during each day of the Morris water maze test. Mann–Whitney test was used to determine the difference in platform crossing number between the control and sevoflurane treatment group. There were no missing data for variables of the Morris water maze test (escape latency and platform crossing number). Two-way ANOVA and post-hoc analysis with Bonferroni were used to determine the interaction and difference between groups (e.g., control versus sevoflurane) and treatments (e.g., vehicle versus lithium). Student’s *t* test was used to determine the difference in two-group comparison when data passed a normality test. One-way ANOVA and post-hoc analysis with Bonferroni were used to determine the differences between groups. *P* < 0.05 was considered statistically significant, and significance testing was two-tailed. Adjusted Bonferroni correction *P*-values were calculated by dividing real *P*-values by experiment size, and adjusted *P*-values were reported in the manuscript. The sample sizes were from 3 to 10 in each group in the present study. The behavioral study to determine the effects of sevoflurane on cognitive function of the mice in MWM was replicated three times by repeating the same anesthesia and using the same Morris water maze protocol. The study to determine EVs makers (*N* = 1 in each group) was replicated three times by repeating the same sevoflurane treatment and using the same Western blot analysis. Statistical analysis was conducted using GraphPad Prism software (version 8.0) and SPSS statistics software (version 21.0).

### Reporting summary

Further information on research design is available in the Nature Research Reporting Summary linked to this article.

## Supplementary information

Supplementary Information

Description of Additional Supplementary Files

Supplementary Data 1

Reporting Summary

## Data Availability

The source data can be found in the Supplementary Data 1 and all other data are available from the corresponding author on reasonable request.
