# Supplementary material for: The anesthetic sevoflurane induces tau trafficking from neurons to microglia
Source: Commun Biol. 2021 May 12;4:560. doi: 10.1038/s42003-021-02047-8 (PMC8115254; doi:10.1038/s42003-021-02047-8)
Supplement: Supplementary file 1 — Supplementary Information [file 42003_2021_2047_MOESM1_ESM.pdf]

Supplemental Information

The anesthetic sevoflurane induces tau trafficking from neurons to microglia

Yuanlin Dong, Feng Liang, Lining Huang, Fang Fang, Guang Yang,
Rudolph E. Tanzi, Yiying Zhang, Qimin Quan and Zhongcong Xie

Supplemental Figure 1.

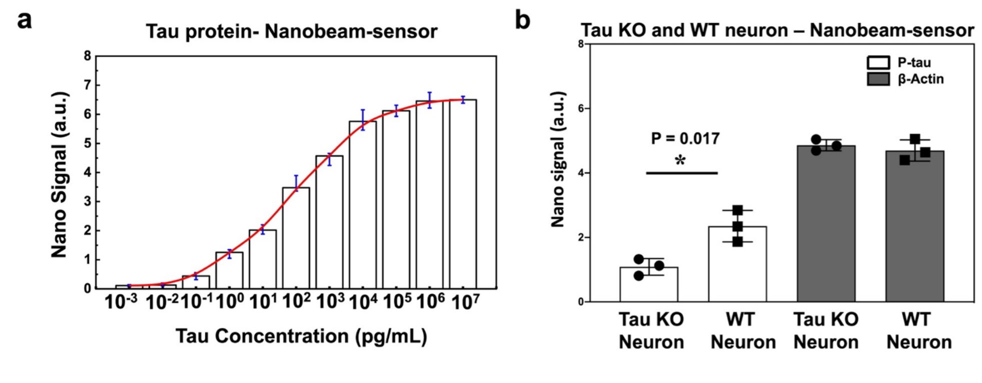

Supplemental Figure 1. Specificity and sensitivity of tau and p-tau measurement by nanobeam-sensor.

a. Different concentrations of tau were prepared and measured by nanobeam-sensor. The different concentrations of tau were associated with different strengths of nanobeam-sensor signal. **b**. Nanobeam-sensor measurement of the p-tau showed that there were higher amounts of p-tau, but not β-Actin, in the WT neurons than those in the tau KO neurons. The signal representing p-tau in tau KO neurons was the background of the measurement. These data suggest that nanobeam-sensor has specificity and sensitivity in detecting tau and p-tau. N = 3 biologically independent samples in each group. The Student's t-test was used to analyze the data presented in supplemental figure 1b, the P value refers to the difference in p-tau amount between the WT neurons and the tau KO neurons, (* = $P < 0.05$). Error bar indicates standard deviation. P-tau, phosphorylated tau; WT, wild-type; tau KO, tau knock out.

Supplemental Figure 2.

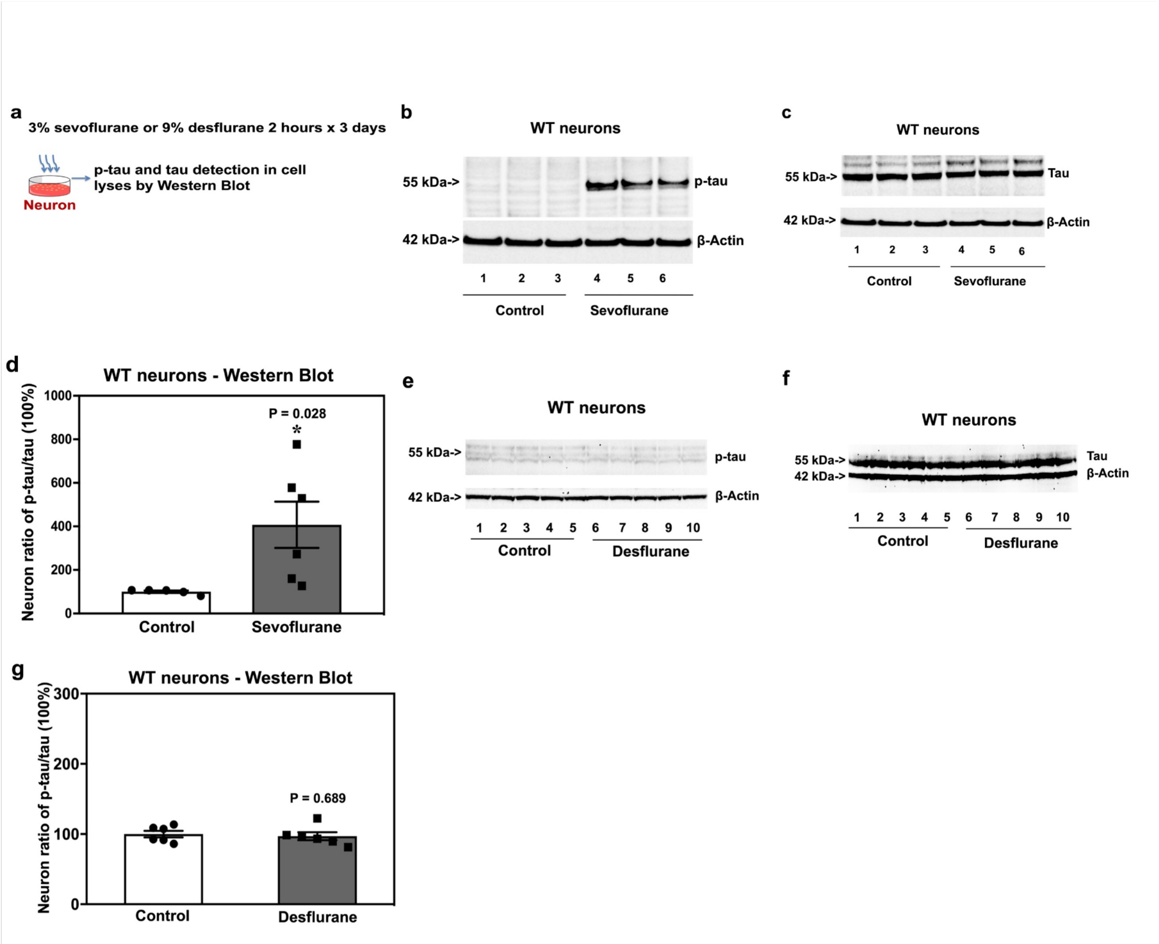

Supplemental Figure 2. Sevoflurane, but not desflurane, induced tau phosphorylation in mouse neurons.

a. The diagram of the experimental design. **b**. Sevoflurane increased p-tau amounts in the lysis of WT mouse neurons. **c**. Effects of sevoflurane on total tau amounts in the lysis of WT mouse neurons. **d**. Quantification of the ratio of p-tau to tau. **e**. Desflurane did not significantly increase p-tau amounts in the lysis of WT mouse neurons. **f**. Effects of desflurane on tau amount in the lysis of WT mouse neurons. **g**. Quantification of the ratio of p-tau to tau. N = 5 or 6 biologically independent samples in each group as demonstrated in the panel of the figure. The Student's t-test was used to analyze the data presented in supplemental figure 2d and 2g, the P values refer to the difference in p-tau/tau ratio between the control condition and sevoflurane or desflurane. * = $P < 0.05$. Error bar indicates standard deviation. P-tau, phosphorylated tau; WT, wild-type.

Supplemental Figure 3.

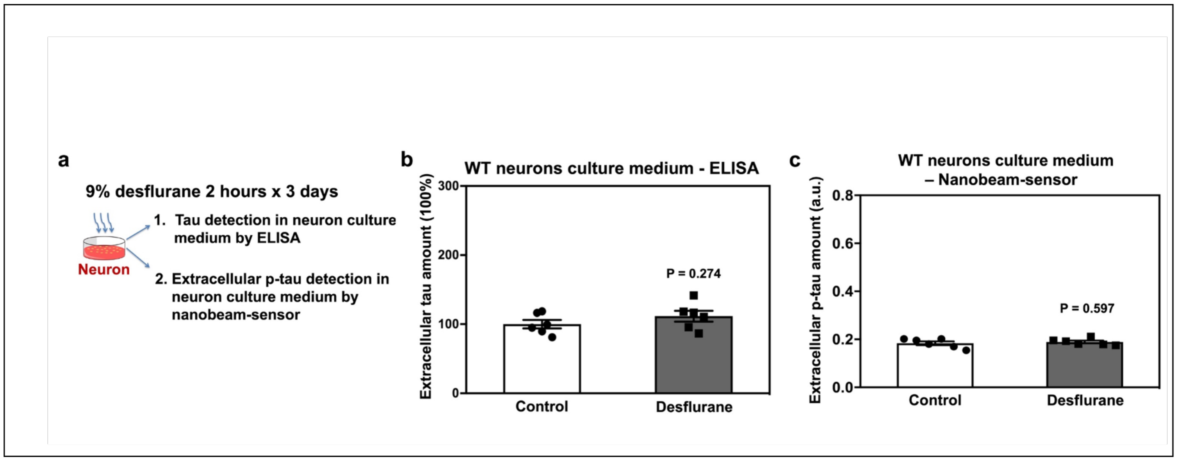

Supplemental Figure 3. Desflurane did not cause exit of tau and p-tau from neurons. **a.** Experimental design. Tau and p-tau outside of neurons in the neuron culture medium were measured by ELISA or nanobeam-sensor after control condition or treatment of desflurane. **b**. ELISA demonstrated that desflurane did not increase the tau amounts outside of neurons in the neuron culture medium as compared to the control condition. **c**. Nanobeam-sensor measurement of p-tau showed that desflurane did not increase the amounts of p-tau outside of neurons in the neuron culture medium as compared to the control condition. N = 6 biologically independent samples in each group. The Student's t-test was used to analyze the data presented in supplemental figure 3b and 3c, the P values refer to the differences in extracellular tau or p-tau amounts between the control condition versus desflurane. Error bar indicates standard deviation. P-tau, phosphorylated tau; WT, wild-type.

Supplemental Figure 4.

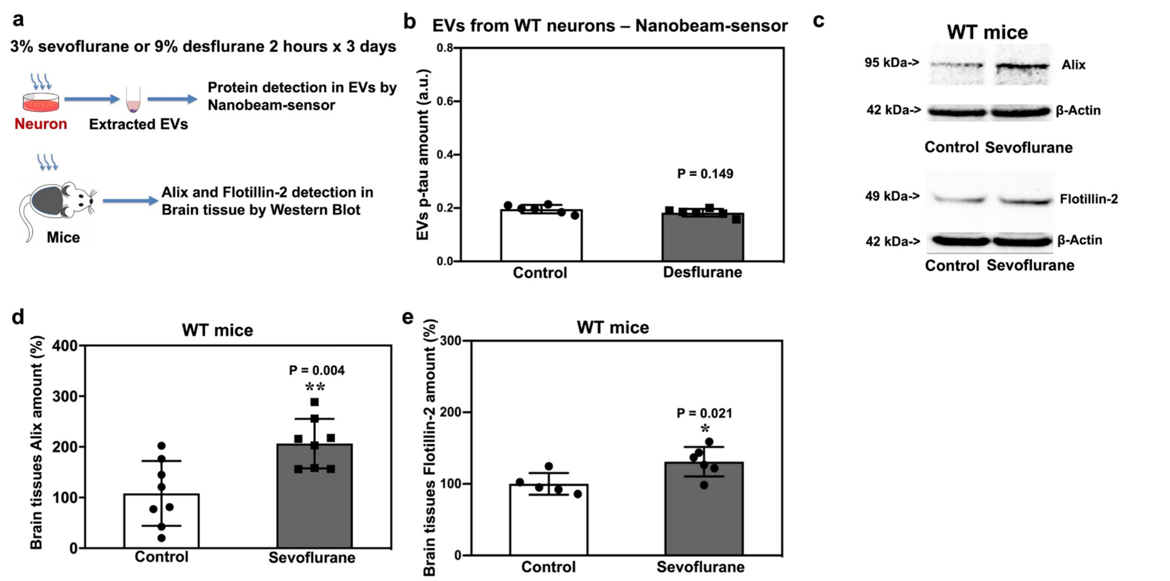

Supplemental Figure 4. Sevoflurane increased the amounts of EVs markers in brain tissues of young mice and desflurane did not increase p-tau amounts in EVs.
a. The diagram of the experimental design. **b**. Nanobeam-sensor determination showed that desflurane did not increase the p-tau amounts in the lysis of EVs extracted from WT neurons as compared to control condition. **c**. Western blot demonstrated that sevoflurane increased the amounts of Alix and Flotillin-2 in the brain tissues of the mice as compared to control condition. **d**. Quantification of Alix. **e**. Quantification of Flotillin-2. N = 5 to 8 independent experiment (Alix and Flotillin-2 study) and 6 biologically independent samples (nanobeam-sensor study in EVs) in each group as demonstrated in the panel of the figure. The Student's t-test was used to analyze the data presented in supplemental figure 4b, 4d and 4e, the P values refer to the difference in the amounts of p-tau, Alix and flotillin-2 between the control condition and sevoflurane or desflurane. * = P < 0.05; ** = P < 0.01. Error bar indicates standard deviation. P-Tau, phosphorylated Tau; WT, wild-type; EVs, extracellular vesicles.

Supplemental Figure 5.

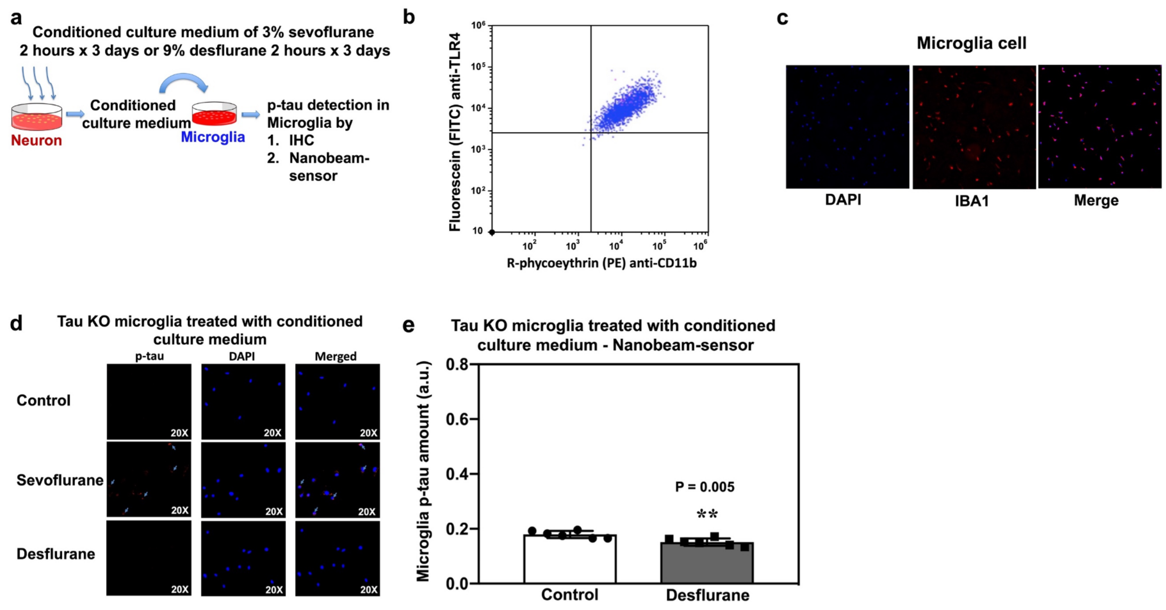

Supplemental Figure 5. Sevoflurane-conditioned culture medium of neurons, but not desflurane-conditioned culture medium of neurons, caused appearance of p-tau in tau KO microglia.

a. The diagram of the experimental design. **b**. The identification of microglia by flow cytometry. **c**. The identification of microglia by immunohistochemistry. **d**. Immunohistochemistry of p-tau in the tau KO microglia following control condition or the administration of sevoflurane or desflurane. **e**. Nanobeam-sensor determination showed that desflurane did not increase the amounts of p-tau in the lysis of the tau KO microglia as compared to control condition. N = 6 biologically independent samples (tau KO microglia studies) in each group. The Student's t-test was used to analyze the data presented in supplemental figure 5e, the P value refers to the difference in p-tau amounts between the control condition and desflurane. ** = $P < 0.01$. Error bar indicates standard deviation. P-Tau, phosphorylated tau; KO, knockout.

Supplemental Figure 6.

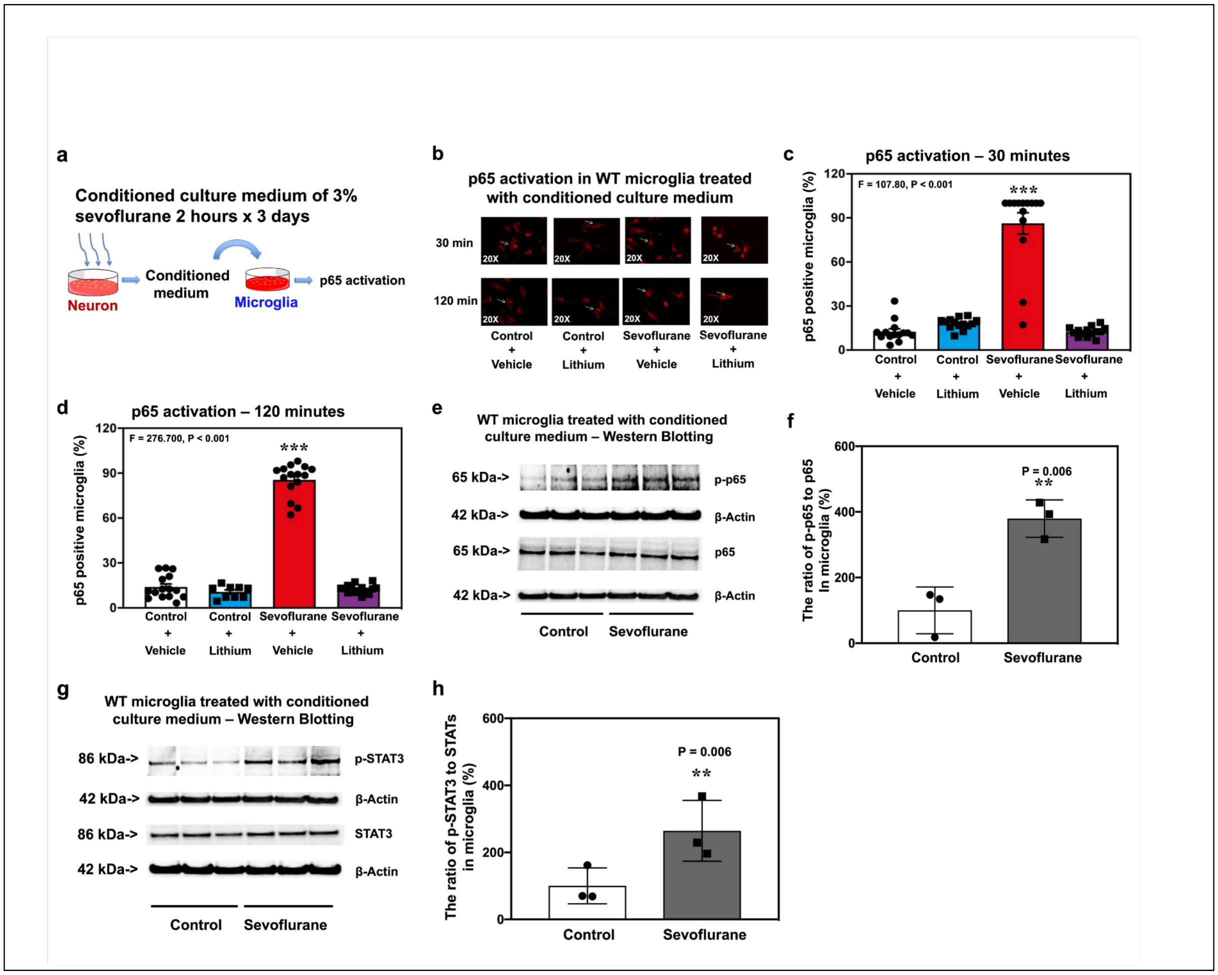

Supplemental Figure 6. Sevoflurane induced activation of p65 and STAT3 in WT microglia.

a. The diagram of the experimental design. Immunohistochemistry determination of the effects of sevoflurane-conditioned culture medium of neurons on p65 activation in the WT microglia for 30 or 120 minutes. **b**. Immunohistochemistry of p65 inside nucleus. **c**. Quantification of p65 activation for 30 minutes. **d**. Quantification of p65 activation for 120 minutes. **e**. Western blot analysis of effects of sevoflurane on the amounts of p65 and p-p65. **f**. Quantification of e. **g**. Western blot analysis of effects of sevoflurane on the amounts of STAT3 and p-STAT3. **h**. Quantification of g. N = 3 biologically independent samples in each group western blot study. N = 15 biologically independent samples in each group in p65 imaging study. Two-way ANOVA and post-hoc analysis with Bonferroni were used to analyze the data presented in supplemental figure 6c and 6d, the P values refer to the interaction of group (control condition versus sevoflurane) and treatment (vehicle versus lithium) on p65 positive microglia. The P values of post-hoc analysis with Bonferroni refer to the difference on p65 positive microglia between the control condition versus sevoflurane. Student's t-test was used to determine the difference of the ratios of phosphorylated p65 to p65 (Supplemental figure 6f) and phosphorylated STAT3 to STAT3 (Supplemental figure 6h), p values refer to the difference in the ratios between control condition and sevoflurane. ** = $P < 0.01$; *** = $P < 0.001$. Error bar indicates standard deviation. WT, wild-type; p-p65, phosphorylated p65; p-STAT3, phosphorylated STAT3.

Supplemental Figure 7.

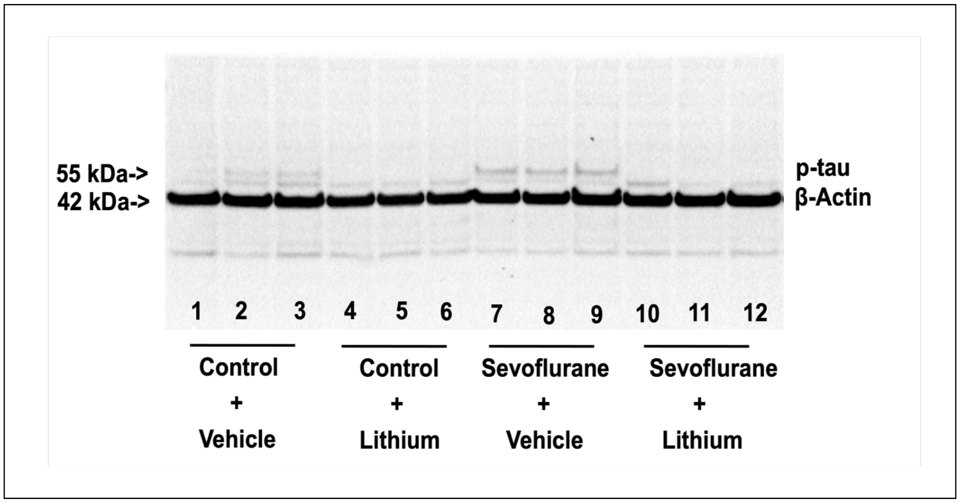

Supplemental Figure 7. Effects of sevoflurane on p-tau in neuron lysates.
This is the full and uncropped blot/gel image for the Figure 2b in the main text, indicating that sevoflurane induced tau phosphorylation in neuron lysates, which was attenuated by lithium. P-tau, phosphorylated tau.

Supplemental Figure 8.

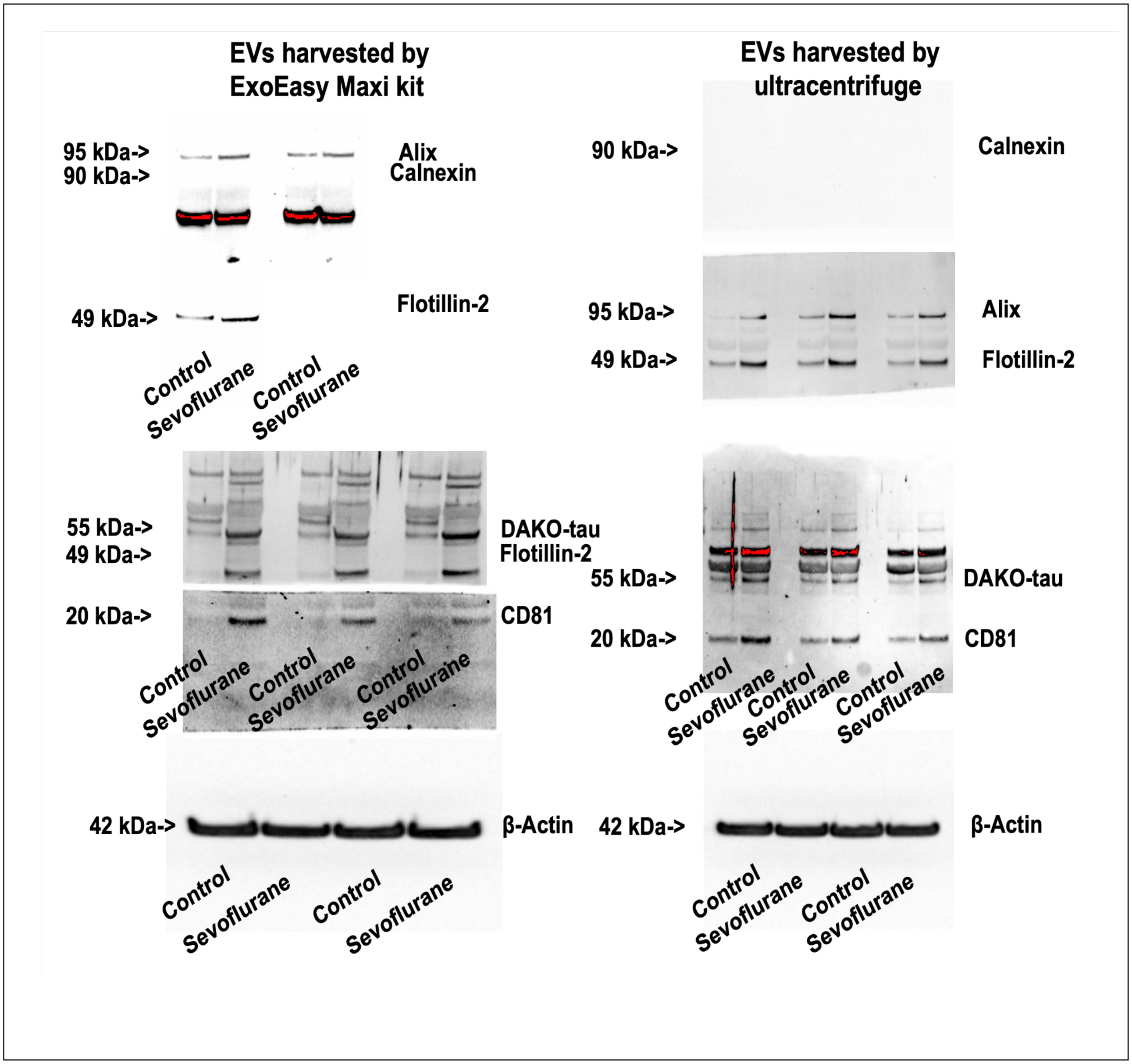

Supplemental Figure 8. Effects of sevoflurane on the markers of EVs and tau in EVs. These are the full and uncropped blot/gel images for the Figure 3b in the main text, indicating the effects of sevoflurane on the amounts of the markers of EVs and tau in EVs. The blot/gel images of β-Actin were obtained from neurons to demonstrate that the similar amounts of neurons were used to harvest EVs. EV, extracellular vesicle.
