# Supplementary material for: The anesthetic sevoflurane induces tau trafficking from neurons to microglia
Source: Commun Biol. 2021 May 12;4:560. doi: 10.1038/s42003-021-02047-8 (PMC8115254; doi:10.1038/s42003-021-02047-8)
Supplement: Supplementary file 2 — Description of Additional Supplementary Files [file 42003_2021_2047_MOESM2_ESM.pdf]

Description of Additional Supplementary Files

File name: Supplemental Data 1

Description: The source data for Figures 1 to 6 and Supplemental Figures 1 to 6.
